# Measuring the electrical charge and settling velocity of volcanic ash with a new, cost-effective apparatus (VoltAsh) both in the laboratory and in the field

**DOI:** 10.1007/s00445-026-02027-y

**Published:** 2026-08-26

**Authors:** Allan Fries, Eduardo Rossi, Jonathan P. Merrison, Jens Jacob Iversen, Tatsushi Matsuyama, Frédéric Arlaud, Jonathan Lemus, Simon Thivet, Carolina Diaz-Vecino, Riccardo Simionato, Costanza Bonadonna

**Affiliations:** 1 Département des Sciences de la Terre, Université de Genève, Genève, Switzerland; 2 Department of Physics and Astronomy, Aarhus University, Aarhus, Denmark; 3 Department of Science and Engineering for Sustainable Innovation, Soka University, Tokyo, Japan; 4 Département d’informatique, Université de Genève, Carouge, Switzerland

**Keywords:** Volcanic ash, Electrical charges, Particle aggregation, Field apparatus, Sakurajima volcano, Etna volcano, Laboratory experiments, Tephra sedimentation

## Abstract

Electrical charges play a key role in particle-particle interactions during transport and deposition of volcanic ash with significant impact on their atmospheric residence time, as they can influence aggregation processes. Nonetheless, direct field measurements are rare due to logistic challenges, resulting in a very limited understanding of charge generation and aggregation mechanisms. This study introduces VoltAsh, a cost-effective, portable device for measuring the charge and velocity of settling volcanic ash particles using a Through-Type Faraday Cage (TTFC) and a charge amplifier circuit. Laboratory validation confirmed the accuracy of both charge and velocity measurements when compared with state-of-the-art techniques. Field deployments at Sakurajima volcano (Japan) in November 2023 enabled automated, semi-continuous measurements over several hours, detecting particle charges ranging from 0.5 to 250 pC and velocities up to 5 ms$^{-1}$. Measurements at Etna volcano (Italy) in July 2024 detected fewer charged particles, likely due to differences in eruptive style, grain size, particle properties and atmospheric conditions. These findings highlight VoltAsh’s potential for advancing studies of volcanic ash transport and deposition processes.

**Supplementary Information:**

The online version contains supplementary material available at https://doi.org/10.1007/s00445-026-02027-y.

## Introduction

The electrification of volcanic particles (i.e., tephra) during eruptions results from various physical processes. These include fracto-emission, where charge is generated by particle fracturing (James et al. 2000), notably during magma fragmentation, and tribo-electrification, whereby particle collisions produce charge (Alois et al. 2018; Cimarelli et al. 2014). Whilst the processes that control charge generation and segregation in volcanic plumes are not yet fully understood, many studies have advanced our understanding of electrification-related phenomena, including volcanic lightning (Behnke et al. 2013; James et al. 2008; Mather and Harrison 2006; Mcnutt and Thomas 2015; McNutt and Williams 2010). In particular, such lightning observations offer promising opportunities for monitoring volcanic activity (Behncke et al. 2014; Ichihara et al. 2023; Jarvis et al. 2024; Prata et al. 2020; Schultz et al. 2020; Van Eaton et al. 2023).

Volcanic ash (i.e., tephra with diameter < 2 mm) is considered the primary charge carrier in volcanic plumes (Cimarelli and Genareau 2022), and its electrification is thought to play an important role in aggregation processes. During aggregation, particles collide and stick together, forming structures (i.e., aggregates) that are larger than individual volcanic ash particles (e.g., Brown et al. 2012 and references therein). Aggregation may be driven by capillary forces and liquid bridges in wet environments, leading to the formation of accretionary pellets (APs), which are typically spherical to oval and may exhibit layered structures (Mueller et al. 2016; Telling et al. 2013; Van Eaton et al. 2012; Tomita et al. 1985). In dry settings, electrostatic forces can promote the formation of particle clusters (PCs), which are characterised by more irregular morphologies and internal structures (Brown et al. 2012; Vecino et al. 2022; Sorem 1982). For the latter, the presence of charges is expected to modulate both collision rate and sticking efficiency (James et al. 2003; Schumacher 1994; Pollastri et al. 2021). Since aggregation is known to affect the particle settling velocity due to changes in the size and density of the particles (Bagheri and Bonadonna 2016a; Vecino et al. 2022), it has an effect on the transport and sedimentation of volcanic ash (Rose and Durant 2011; Rossi et al. 2021). Fine volcanic ash particles <63μ very often settle as aggregates, causing premature deposition from the ash cloud (Gouhier et al. 2019). It is therefore crucial to quantify the charge carried by volcanic ash particles in order to study aggregation processes and, ultimately, better parametrise their effect on ash sedimentation (Beckett et al. 2022).

Even though particles and aggregates individually carry small electrical charges, typically on the order of 0.1 to 10 pC, measuring these charges is crucial for understanding both micro-scale aggregation processes and macro-scale electrification dynamics within volcanic plumes. At the particle scale, direct field measurements provide fundamental constraints for theoretical and experimental models of charging and aggregation (e.g., Pollastri et al. 2021; Telling and Dufek 2012). At the plume scale, *in-situ* charge measurements on individual particles complement remote sensing techniques by resolving the spatial and temporal distribution of charge (Aplin et al. 2016), assessing charge segregation processes (Miura and Koyaguchi 2002), and investigating how aggregation affects the bulk electrical behaviour of volcanic plumes (Pollastri et al. 2022). For instance, Miura and Koyaguchi (2002) proposed a plume-scale charge structure in which negatively charged fine particles and positively charged coarse particles segregate, potentially producing vertically stratified charge and size distributions, whilst (Pollastri et al. 2022) showed that particle clusters are composed of oppositely charged constituents bonded by attractive electrostatic forces. These findings suggest that electrical forces may influence both aggregation efficiency and the spatial organisation of particles within plumes, with implications for plume evolution and ash transport.

Only few field experiments have directly measured the charge of volcanic ash particles during fallout, highlighting the need for more practical *in-situ* techniques. This is partly due to the lack of sensors adapted to monitor the electrical properties of volcanic ash *in situ* (Cimarelli and Genareau 2022). In fact, as well described in Pollastri et al. (2022), the scarcity of field measurements stems from the complexity of the required techniques, the unpredictability of volcanic activity, and challenging environmental conditions. Previous measurements relied either on Faraday cups connected to electrometers (Gilbert et al. 1991; Hatakeyama 1958), or on the deviation of the trajectories of volcanic ash particles in an electric field (Gilbert et al. 1991; Miura and Koyaguchi 2002; Pollastri et al. 2022). However, only (Pollastri et al. 2022) were able to measure both the bulk charge of aggregates and that of their constitutive particles during fallout, using high-speed imaging of particles settling between two high-voltage copper plates at Sakurajima volcano, Japan. They reported aggregate charges between $10^{-3}$ and 2 pC, whereas fragments of aggregates carried higher charges, between 0.1 and 10 pC, with opposing polarities. When normalised by surface area or mass, these values were consistent with previous measurements by Hatakeyama (1958); Gilbert et al. (1991), and Miura and Koyaguchi (2002). Despite its sensitivity to very low charges, down to the order of $10^{-3}$ pC, the method of Pollastri et al. (2022) was limited to particles larger than 200 μm and to short, discrete recording intervals imposed by high-speed video acquisition. Together with the complexity of the apparatus and its sensitivity to environmental conditions, these limitations make the approach difficult to deploy routinely during eruptions and motivate the development of new *in-situ* methods capable of measuring the charge of individual ash particles and aggregates over longer durations.

The settling velocity of volcanic ash particles is another critical parameter for understanding tephra transport and sedimentation, and it can be obtained alongside electrical charge measurements. It controls the residence time of volcanic ash in the atmosphere, thereby influencing airborne concentrations, dispersal, and deposition patterns, with implications for hazard and risk assessments (Bonadonna et al. 2021; Jenkins et al. 2015). Ash transport and dispersal models rely on formulations of the particle settling velocity based on particle properties, including size, density, and shape (Bonadonna and Costa 2013; Folch et al. 2009, 2020; Heffter and Stunder 1993). Direct *in-situ* measurements contribute to a better understanding of the contribution of particle characteristics and atmospheric properties on settling velocity (Freret-Lorgeril et al. 2019; Iguchi et al. 2019). Common instruments, such as high-speed cameras (Bagheri and Bonadonna 2016a; Gabellini et al. 2020; Miwa et al. 2020; Vecino et al. 2022) and optical disdrometers (Freret-Lorgeril et al. 2022; Kozono et al. 2019; Scollo 2005; Suh et al. 2019; Takishita et al. 2024), provide useful measurements but are limited to specific particle size and velocity ranges. Combining complementary sensors can address these limitations and ultimately improve the characterisation of eruptions and associated fallout (Freret-Lorgeril et al. 2021).

Here, we first describe VoltAsh, a cost-effective device inspired by laboratory apparatuses (Méndez Harper and Dufek 2016; Watanabe et al. 2006, 2007) and designed to measure the electrical charge and fall velocity of volcanic ash particles *in situ* over long durations and at high temporal resolution. We then present laboratory experiments to test the device under controlled conditions and validate it against independent measurements. Finally, we report field measurements from Sakurajima and Etna volcanoes, estimate the electrical charge of ash particles settling from volcanic plumes, and discuss implications for future studies of plume electrification and charge-dependent aggregation processes.

## Methods

### Principles of charge and velocity measurements

Charge and velocity measurements are performed using Through-Type Faraday Cages (TTFCs), a modified form of Faraday cages that allows the charge of airborne objects to be measured. TTFCs connected to charge amplifiers have been widely used in laboratory experiments to measure the electrical charge of individual falling or colliding particles (Matsusaka et al. 2010; Méndez Harper and Dufek 2016; Méndez Harper et al. 2018, 2021, 2020; Naik et al. 2016; Watanabe et al. 2006, 2007). These measurements have notably been applied to quantify charge transfer during impact (Watanabe et al. 2006, 2007), tribo-electrification of volcanic ash (Méndez Harper and Dufek 2016; Méndez Harper et al. 2020, 2021), and electrification processes in extraterrestrial analogue materials (Méndez Harper et al. 2018).

**Fig. 1:**
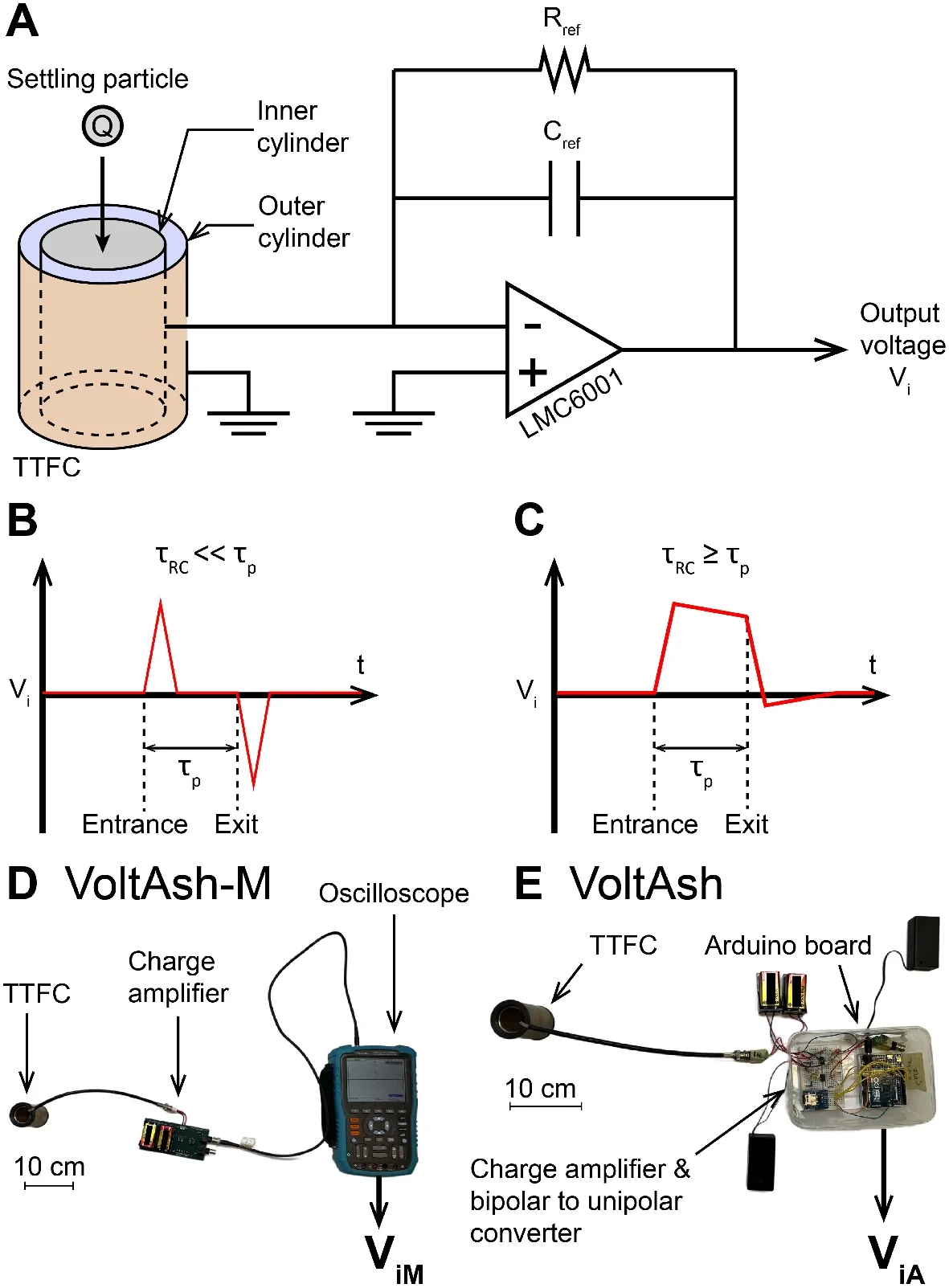
**A** Schematic representation of the Through-Type Faraday Cage (TTFC) connected to the charge amplifier circuit. **B** Illustrative voltage signal generated by the passage of a negatively charged object across the TTFC for $\tau _\text {RC} \ll \tau _\text {p}.$ **C** $\tau _\text {RC} \ge \tau _\text {p}$. **D** Pictures of the instruments and TTFCs. Version operated manually with an oscilloscope (VoltAsh-M). **E** Autonomous version controlled with an Arduino board (VoltAsh)

The main components of the TTFC and charge amplifier are shown in Fig. 1A. A TTFC consists of two coaxial metal cylinders separated by insulating spacers. The outer cylinder is grounded to shield the sensor from electrical interference, whereas the spacers limit charge leakage (Naik et al. 2016). When an object carrying a charge *Q* enters the TTFC, it induces an electron flow on the shielded inner cylinder (Weinheimer 1988). This cylinder is connected to a charge amplifier, which integrates the induced current using a feedback capacitor $C_\text {ref}$ and an ultra-low input current operational amplifier (such as the LMC 6001 by Texas Instruments; Méndez Harper et al.^2018^; Watanabe et al. 2007). The output voltage $V_\text {i}$ is proportional to the charge of the object and is given by

1 $$\begin{aligned} V_\text {i} = -\frac{Q}{C_\text {ref}}. \end{aligned}$$

A charged object therefore produces a voltage peak whose amplitude and sign respectively depend on the magnitude and polarity of *Q* (Eq. 1; Watanabe et al. 2006, 2007). Similar apparatuses have measured bulk charges as low as $10^{-3}$ pC (Méndez Harper and Dufek 2016; Méndez Harper et al. 2020; Watanabe et al. 2007).

Afterward, when the charged object leaves the TTFC, an inverse electron flow is induced on the inner cylinder. If the circuit response is sufficiently fast, this produces a second, symmetric voltage peak of opposite sign (Fig. 1B). Whilst the gain of the charge amplifier is set by $C_\text {ref}$, its response time is controlled by the time constant $\tau _\text {RC}$, defined by the feedback resistance $R_\text {ref}$ and capacitance $C_\text {ref}$:

2 $$\begin{aligned} \tau _\text {RC} = R_\text {ref} \times C_\text {ref}. \end{aligned}$$

Two symmetric peaks can be resolved when $\tau _\text {RC}$ is much shorter than the object residence time $\tau _\text {p}$ within the TTFC. Assuming that the object falls at its terminal velocity, $\tau _\text {p}$ is related to the TTFC length *L* and object velocity *v* by

3 $$\begin{aligned} \tau _\text {p} = \frac{L}{v}. \end{aligned}$$

Otherwise, if $\tau _\text {RC} \ge \tau _\text {p}$, the entrance and exit signals overlap. In this case, the first voltage peak decays exponentially, at a rate controlled by $\tau _\text {RC}$, before being abruptly dampened by the inverse current generated as the object exits the TTFC (Fig. 1C).

It is therefore possible to estimate $\tau _\text {p}$ from the analysis of the voltage signal and, given *L*, *v* can be determined from Eq. 3. The parallel application of TTFCs for measuring *v* is documented in several studies (e.g., the “dynamic Faraday cups” in Kucerovsky and Kucerovsky 2003; MacLatchy 1988), but is rarely used for relatively slow objects for which $\tau _\text {RC} \ll \tau _\text {p}$.

The TTFC geometry also affects the accuracy of charge measurements. Weinheimer (1988) demonstrated that the aspect ratio of the TTFC, defined as the inner radius divided by the half-length of the cage, controls its ability to measure *Q*. To achieve sufficiently low uncertainties, the aspect ratio should be ≥ 1. Consequently, TTFCs are generally designed to be at least twice as long as the aperture diameter (Méndez Harper and Dufek 2016; Watanabe et al. 2007).

### Instrumental design and operation

Two versions of the same instrument, VoltAsh-M and VoltAsh, were developed to measure electrical charge and settling velocity based on the principles described in the “Principles of charge and velocity measurements” section. In both versions, $R_\text {ref}$ is fixed at 1 G${\Omega }$, and $C_\text {ref}$ can be set to either 10 or 100 pF, depending on the expected magnitude of *Q*. These capacitances yield $\tau _\text {RC}$ values of 10 or 100 ms, respectively. Both versions use charge amplifiers built around an LMC6001 operational amplifier.

VoltAsh-M was designed for short-duration, high resolution, manually triggered measurements. In this version, the charge amplifier is mounted directly on a printed circuit board (Supplementary Fig. S1) similarly to Méndez Harper et al. (2021), and the output voltage is recorded with a SHS820 handheld digital oscilloscope (Fig. 1D). VoltAsh-M enables punctual measurements, with voltage recordings limited to less than 1 s at a typical sampling frequency of 50 kHz and a resolution of 1 mV. Manual operation is required to trigger measurements and save data. The charge amplifier is powered by 9 V batteries, producing an output voltage $V_\text {iM}$ between −9 and 9 V.

VoltAsh was designed as an autonomous version for longer field deployments. It uses an Arduino UNO R4 Minima board to read the output voltage and save the acquired data (Fig. 1E). Arduino microcontrollers have previously been used in geosciences (Andò et al. 2015; Guerriero et al. 2017; Idehen and You 2023), illustrating their versatility. Although its sampling rate of 0.4–1 kHz is lower than that of VoltAsh-M, it is sufficient for almost continuous measurements over long durations. Because the ARM Cortex-M4 processor of the Arduino board is single-threaded, data cannot be read and saved simultaneously. Voltage acquisition is therefore performed in blocks of 750 samples, corresponding to a maximum duration of 2 s, separated by saving intervals of up to 0.5 s, during which data are written to an SD card.

VoltAsh includes a voltage conversion stage to make the charge-amplifier output compatible with the Arduino’s analogue-to-digital converter (ADC). As described above, the charge amplifier outputs voltages between −9 and 9 V. A bipolar-to-unipolar converter was therefore added in series with the charge converter to transform this signal into an output voltage $V_\text {iA}$ between 0 and 5 V, which can be read by the ADC (Supplementary Fig. S2). This converter compresses the signal by a factor *f* of 3.6 and adds an offset voltage ΔV of approximately 2.5 V. Thus, input voltages of −9, 0, and 9 V are converted to 0, 2.5, and 5 V, respectively. The particle charge is then calculated as

4 $$\begin{aligned} Q = -f(V_\text {iA} - \Delta V) C_\text {ref}. \end{aligned}$$

The ADC resolution was set to 14 bits, providing a voltage sensitivity of 1 mV, equivalent to 3.6 mV at the charge-amplifier output.

The TTFCs were designed to be compatible with both versions of the instrument. They were built from stainless-steel or brass cylinders with variable lengths *L* and aperture diameters *D* (Supplementary Fig. S3). All TTFCs have an aspect ratio ≥ 2. The outer and inner cylinders are 5 mm thick and separated by 5 mm insulating spacer rings.

Both versions were assembled from inexpensive and accessible components to facilitate reproduction. The printed circuit board of VoltAsh-M was produced at the Haute École du Paysage, d’Ingénierie et d’Architecture of Geneva, the stainless-steel TTFCs at Aarhus University, and the brass TTFCs and VoltAsh at the University of Geneva. VoltAsh is particularly low-cost, with a total component cost of approximately 80 US dollars, and is straightforward to assemble. This simple and accessible design makes the instrument suitable for scientific research, monitoring, teaching, and outreach activities. A detailed schematic of the circuit and Arduino connections is provided in Supplementary Fig. S4, and the Arduino code (i.e., “sketch”, the standard term for an Arduino program) used to read voltage and save data to the SD card is provided in Supplementary Data S1.

**Fig. 2:**
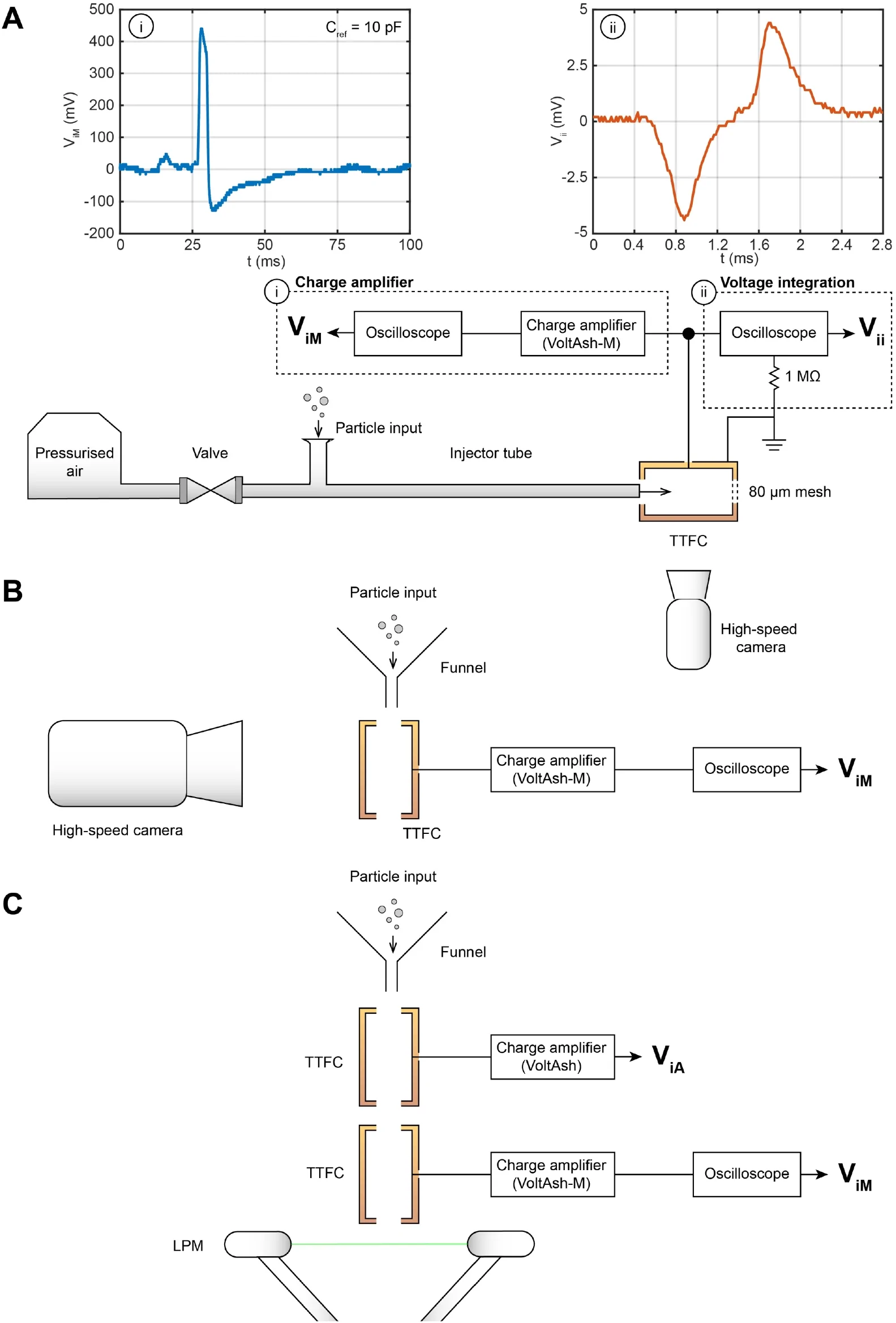
Experimental setups used to validate particle charge and velocity measurements. **A** Comparison between charge measurements obtained with (i) VoltAsh-M and (ii) direct voltage integration. The areas labelled (i) and (ii) indicate the two charge measurement techniques. Examples of typical voltage outputs are shown for (i) Ex18: $C_\text {ref}$ = 10 pF, $R_\text {ref}$ = 1 GΩ, *d* = 2639 ± 92 μm, *L* = 128 mm; and (ii) Ex14: $R_\text {d}$ = 1 GΩ, *d* = 2639 ± 92 μm, *L* = 40 mm (Table 1). In some experiments, particle velocities were obtained from both the voltage signal and high-speed camera images. **B** Simultaneous velocity measurements using VoltAsh-M and a high-speed camera for particles falling across the TTFC. **C** Validation of charge measurements with VoltAsh and comparison between particle settling velocities estimated by VoltAsh, VoltAsh-M, and an LPM

### Laboratory experiments

We conducted laboratory tests to benchmark particle charge measurements obtained with VoltAsh-M. The tests were performed at the Planetary Environmental Facility of Aarhus University as part of a Europlanet Transnational Access Programme (TA2.4 - PEF #22-EPN3-060), using an experimental setup adapted from Alois et al. (2018) and Alois (2019). They involved decompressing small volumes of pressurised air through an aluminium oxide (Al_2_O_3_) tube containing either a small mass of fine silica beads or larger individual glass beads (Fig. 2A; Table 1). The fine particles used in experiments Ex1–Ex12 (Table 1) had a median equivalent diameter of 118.3 μm and a sorting coefficient of 30.6 μm (Inman 1952), as measured from Celestron handheld digital microscope images (Supplementary Fig. S5). Microscope images were also used to measure the Feret diameters of the larger glass beads reported in Table 1 (Ex14, Ex15, Ex18 and Ex19). Additional experiments were performed using volcanic ash from Sakurajima volcano (Ex16; Table 1) and quartz crystals (Ex13 and Ex17; Table 1).

**Table 1:** List of laboratory experiments conducted to validate particle charge measurements. Only experiments Ex20 and Ex21 were conducted specifically for validating velocity measurements (Fig. 2B, C). *d*, *m*, and *Q* are the median diameter, the mass of particles dispersed, and the bulk particle charge, respectively. Mean values of *m* and *Q* are given, with standard deviation in brackets. The sorting coefficient of Inman (1952) is given in brackets for fine particles. Individual particles are characterised by their mean Feret diameter, with deviation to the maximum Feret diameter in brackets. Glass beads were used in experiments Ex1–Ex12, Ex14–Ex15 and Ex18–Ex20, whilst quartz crystals were used in Ex13 and Ex17, and volcanic ash in Ex16. Glass beads and volcanic ash were used in Ex21. A more detailed list, including information on repeat experiments, voltage signals, and high-speed videos, can be found in Supplementary Data S2

Experiment number	Number of repeats	Measurement technique	d (μm)	*m* (mg)	|*Q*| (pC)
Ex1	4	Voltage integration ($R_\text {d} = 1~\text {M}\Omega$)	118.3 (30.6)	0.4 (0.08)	16 (5)
Ex2	5	Voltage integration ($R_\text {d} = 1~\text {M}\Omega$)	118.3 (30.6)	4 (0.8)	37 (7)
Ex3	3	Voltage integration ($R_\text {d}= 1~\text {M}\Omega$)	118.3 (30.6)	8 (1.5)	89 (30)
Ex4	7	Voltage integration ($R_\text {d} = 1~\text {M}\Omega$)	118.3 (30.6)	12 (2)	136 (77)
Ex5	10	Voltage integration ($R_\text {d} = 1~\text {M}\Omega$)	118.3 (30.6)	40 (8)	1590 (1031)
Ex6	9	VoltAsh-M ($C_\text {ref} = 10~\text {pF}$)	118.3 (30.6)	0.4 (0.08)	6 (3)
Ex7	8	VoltAsh-M ($C_\text {ref} = 10~\text {pF}$)	118.3 (30.6)	4 (0.8)	16 (8)
Ex8	10	VoltAsh-M ($C_\text {ref} = 10~\text {pF}$)	118.3 (30.6)	8 (1.5)	23 (8)
Ex9	2	VoltAsh-M ($C_\text {ref} = 10~\text {pF}$)	118.3 (30.6)	12 (2)	31 (4)
Ex10	4	VoltAsh-M ($C_\text {ref} = 100~\text {pF}$)	118.3 (30.6)	4 (0.8)	30 (8)
Ex11	3	VoltAsh-M ($C_\text {ref} = 100~\text {pF}$)	118.3 (30.6)	8 (1.5)	53 (6)
Ex12	3	VoltAsh-M ($C_\text {ref} = 100~\text {pF}$)	118.3 (30.6)	12 (2)	100 (10)
Ex13	12	Voltage integration ($R_\text {d} = 1~\text {M}\Omega$)	1762.5 (108)	individual	5 (5)
Ex14	2	Voltage integration ($R_\text {d} = 1~\text {M}\Omega$)	2639 (92)	individual	2 (1)
Ex15	3	Voltage integration ($R_\text {d} = 1~\text {M}\Omega$)	3891 (24)	individual	12 (9)
Ex16	11	VoltAsh-M ($C_\text {ref} = 10~\text {pF}$)	799 (52)	individual	0.2 (0.1)
Ex17	8	VoltAsh-M ($C_\text {ref} = 10~\text {pF}$)	1762.5 (108)	individual	4 (2)
Ex18	17	VoltAsh-M ($C_\text {ref} = 10~\text {pF}$)	2639 (92)	individual	6 (4)
Ex19	4	VoltAsh-M ($C_\text {ref} = 10~\text {pF}$)	3891 (24)	individual	17 (6)
Ex20	5	VoltAsh-M ($C_\text {ref} = 10~\text {pF}$)	1400 (86)	individual	−
Ex21	9	Voltash-M and VoltAsh ($C_\text {ref} = 10~\text {pF}$)	1400 (86); 830 (120)	individual	15 (9)

This first set of experiments allowed to generate charged particles and compare two charge measurement techniques: (i) VoltAsh-M and (ii) voltage integration. The release of pressurised air, with overpressures of 0.3 to 3 bar, was initiated by electronically opening a valve, which simultaneously triggered voltage measurements. Particles were then dispersed within the 50 cm long injector tube, with a diameter of 0.4 cm, and acquired electrical charges through particle-particle and particle-tube contact electrification. A TTFC was positioned at the exit of the injector tube to measure particle charge either (i) with VoltAsh-M, using Eq. 1, or (ii) by voltage integration of the time-dependent signal $V_{ii}$ generated in the TTFC. For voltage integration, $V_{ii}$ was recorded through time with an oscilloscope, and the charge *Q* of the material in the TTFC was obtained as Alois et al. (2018)

5 $$\begin{aligned} Q = \int \frac{V_{ii}(t)}{R_\text {d}}dt, \end{aligned}$$

with $R_d$ the discharge impedance between the TTFC and the floating ground. The charge surface density σ of the particles was then estimated as

6 $$\begin{aligned} \sigma = \frac{|Q|}{\pi d^2}, \end{aligned}$$

with *d* the particle diameter.

**Table 2:** Field measurements conducted with VoltAsh at Sakurajima and Etna volcanoes. GPS coordinates for each field location are provided in Supplementary Table S1. The feedback impedance $R_\text {ref}$ was fixed at 1 GΩ, and the TTFC length was 58 mm, except for F19, where it was 100 mm. Voltage signals associated with all field measurements are provided in Supplementary Data S3

Field meas. number	Location	Date (dd/MM/yyyy)	Time (hh:mm)	Duration (s)	$C_{\text {ref}}$ (pF)	Number of charged objects analysed
F1	Sk1	10/11/2023	11:13	5603.5	10	1
F2	Sk2	11/11/2023	11:57	1101.1	10	2
F3	Sk2	11/11/2023	14:25	2441.6	10	1
F4	Sk2	12/11/2023	14:38	1428.4	10	3
F5	Sk3	13/11/2023	17:42	8618.0	10	1
F6	Sk4	14/11/2023	10:01	1231.1	10	2
F7	Sk2	15/11/2023	10:55	761.0	10	3
F8	Sk2	15/11/2023	10:55	764.9	100	3
F9	Sk2	15/11/2023	12:04	975.0	10	8
F10	Sk2	15/11/2023	12:04	975.0	100	5
F11	Sk2	15/11/2023	12:50	3139.3	10	4
F12	Sk2	15/11/2023	12:50	3138.9	100	4
F13	Sk2	15/11/2023	16:45	2451.0	10	3
F14	Sk2	15/11/2023	16:45	2458.6	100	2
F15	Sk2	18/11/2023	10:42	1457.3	100	1
F16	Sk2	18/11/2023	10:45	1304.6	10	5
F17	Sk2	18/11/2023	16:44	28078.0	10	2
F18	Et1	16/07/2024	01:24	3005.8	10	2
F19	Et2	23/07/2024	07:22	6293.5	100	0
F20	Et1	23/07/2024	08:24	14552.9	10	3

The two charge measurement techniques were applied in repeat experiments to compare their results. Typical voltage signals obtained using (i) VoltAsh-M and (ii) voltage integration are shown in Fig. 2A. In some experiments, an 80 μm metallic mesh was placed over the TTFC outlet. This setup allowed air to pass through while retaining the particles, thereby facilitating charge measurement. A total of 19 experiments and 125 repeat tests were performed in this configuration (Ex1–Ex19; Table 1). In 13 repeat tests using VoltAsh-M, a Phantom Miro M110 or an Edgertronic high-speed camera was used to film individual particles crossing the TTFC. These images provided independent velocity measurements for comparison with velocities inferred simultaneously from voltage signals. Because particles were released at high speed, these tests evaluated velocity measurements for $\tau _\text {RC} \ge \tau _\text {p}$ (Fig. 1C).

This analysis was complemented by two additional laboratory experiments and 14 repeat tests performed at the University of Geneva to validate both charge measurements with VoltAsh and particle settling velocity measurements. In these experiments, particles settled more slowly, allowing velocity measurements to be tested under conditions where $\tau _\text {RC} \ll \tau _\text {p}$ (Fig. 1B). The first set of experiments used VoltAsh-M only and consisted of filming charged particles settling through a TTFC with a Phantom Miro M110 high-speed camera, in order to compare camera-derived velocities with velocities obtained from VoltAsh-M voltage signals (Fig. 2B; Ex20 in Table 1). The second set of experiments was used to compare charge measurements obtained with VoltAsh and VoltAsh-M. In the same setup, particle settling velocities were estimated simultaneously with VoltAsh, VoltAsh-M, and a Thies Clima Laser Precipitation Monitor (LPM; Fig. 2C; Ex21 in Table 1). The LPM measured the size and settling velocity of objects larger than 0.15 mm and smaller than 10 mm passing through a 22.8 × 2 cm$^{2}$ laser beam (Freret-Lorgeril et al. 2022). Particles were mainly silica glass beads with diameters of 1400 ± 86 μm, charged beforehand by friction in a PVC tube. Volcanic ash particles from Etna volcano, with diameters ranging from 710 to 950 μm, were also used in one repeat experiment (Ex21_9; Supplementary Data S2). All tests were performed at atmospheric pressure, ambient temperature of approximately 20 $^{\circ }$C, and relative humidity of about 50%. The effect of environmental conditions on instrument performance was not evaluated, but is expected to be limited for typical laboratory and field measurements, as similar devices provided reliable measurements at 100% relative humidity (Méndez Harper et al. 2022).

**Fig. 3:**
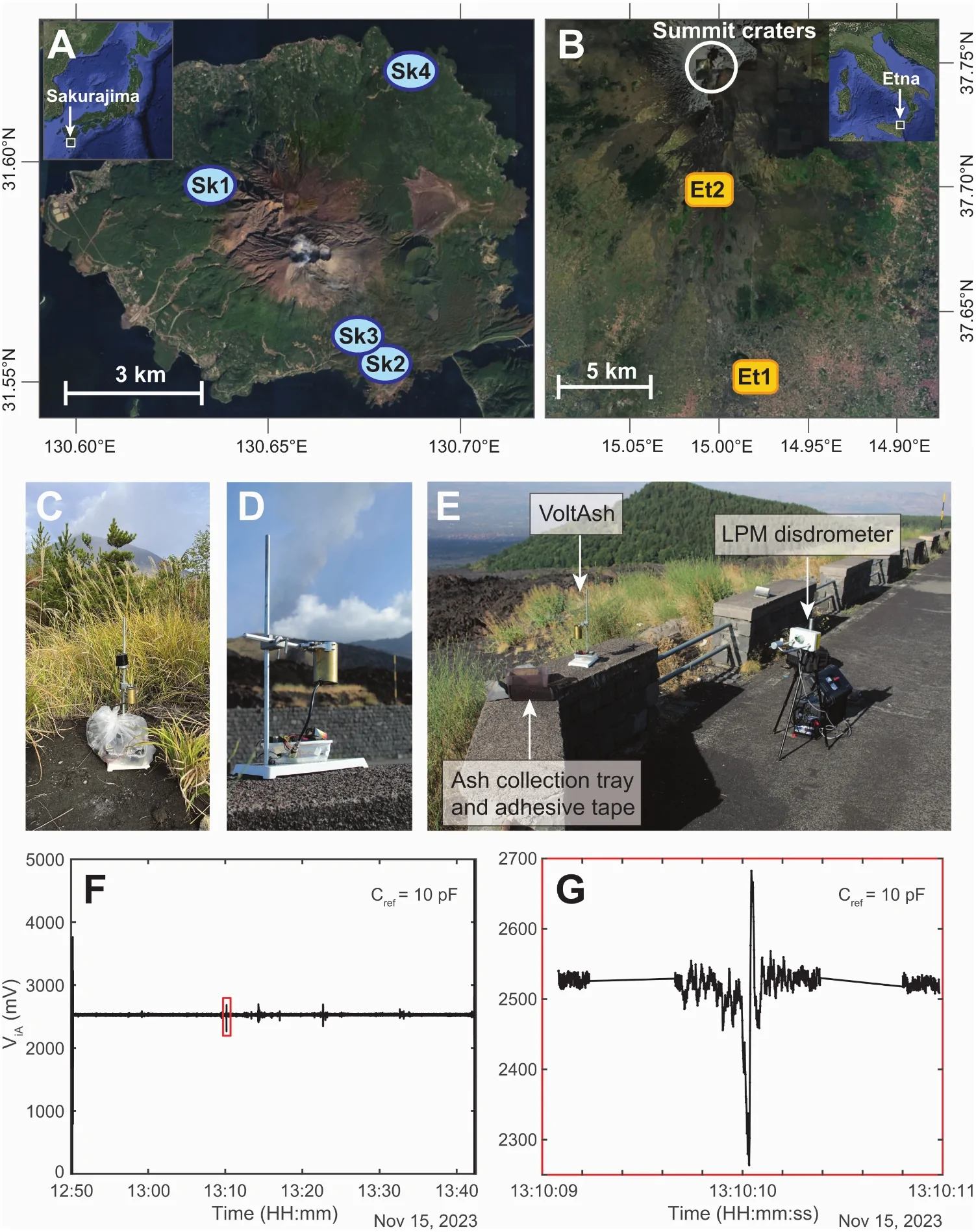
Field locations, instruments, and example VoltAsh signals from the Sakurajima and Etna campaigns. **A** Field sites at Sakurajima volcano in November 2023 and **B** at Etna volcano in July 2024. Labels correspond to the locations listed in Table 2. **C** and **D** Instruments and sampling methods deployed at Sakurajima and Etna, respectively. VoltAsh is shown at location Sk3 at Sakurajima (**C**) and location Et2 at Etna (**D**). **E** Field setup at location Et2, including VoltAsh, the LPM, and the ash collection tray. **F** Voltage signal recorded during field measurement F11 at Sakurajima volcano and **G** close-up of a single voltage peak produced by a charged object crossing the TTFC, corresponding to the red box in **F**

### Field campaigns

VoltAsh was first tested at Sakurajima volcano, Japan, from 7 to 19 November 2023. Sakurajima has produced frequent Vulcanian eruptions since 1955 (Iguchi et al. 2019; Ishizaki et al. 2025), and volcanic lightning has been repeatedly evidenced during its activity (Aizawa et al. 2016; Cimarelli et al. 2016; Smith et al. 2018, 2021). The electrification of volcanic ash particles has also been directly measured *in situ* (Hatakeyama 1958; Miura and Koyaguchi 2002; Pollastri et al. 2022). Electrical charges are expected to influence ash aggregation at Sakurajima, where particle clusters are commonly observed in the sedimentation (Bagheri et al. 2016; Gabellini et al. 2020; Vecino et al. 2022; Miura and Koyaguchi 2002; Gilbert et al. 1991). During the November 2023 campaign, activity was dominated by gas emissions, ash venting, and a few weak eruptions producing plumes lower than 2000 m above the vent (Thivet et al. 2025, 2026). Plume heights were documented by the Japan Meteorological Agency dataset (https://www.jma-net.go.jp/kagoshima/vol/data/skr_exp_list/skr_exp_2023.html) and measured using visible-wavelength cameras calibrated following (Simionato et al. (2022)) and Snee et al. (2023). VoltAsh was deployed at different locations around the volcano, depending on wind direction and plume dispersal axis, to analyse the electrical charge of ash particles and aggregates produced by this weak activity (Table 2; Fig. 3A, C).

VoltAsh was subsequently deployed at Etna volcano, Italy, during the paroxysmal eruptions of 16 and 23 July 2024. Etna frequently produces paroxysmal eruptions associated with lava fountains and ash clouds rising several kilometres into the atmosphere (Andronico et al. 2021; Giuffrida et al. 2023; Scollo et al. 2014). Several paroxysms occurred at the Voragine crater in July 2024, including on 16 and 23 July, when plumes reached approximately 6 and 8 km above sea level, respectively (Global Volcanism Program 2024; Thivet et al. 2025). VoltAsh was deployed at two locations along the main plume dispersal axis (Fig. 3B, D).

The voltage signals recorded by VoltAsh were processed manually to identify charged objects crossing the TTFC (Fig. 3F, G). Voltage peaks with amplitudes clearly exceeding the background noise were selected, while truncated signals, overlapping peaks, and artifacts recorded immediately after the start or before the end of acquisition (i.e., at the start/stop of the Arduino) were discarded. Only peaks resembling the typical signals shown in Figs. 1B, C and 2A were analysed (Supplementary Data S4 and S5). Consequently, the number of charged objects reported in Table 2 underestimates the actual sedimentation rate of charged particles. For each selected peak, the polarity and bulk charge of the object were determined from the sign and amplitude of the voltage signal, respectively. Positive voltage peaks corresponded to negatively charged objects, whereas negative peaks corresponded to positively charged objects. As in the laboratory experiments, the −9 to 9 V output of the charge amplifier was converted to a 0 to 5 V signal using Eq. 4. The mean signal value corresponding to ΔV (∼2.5 V) was therefore subtracted from the peak amplitude, and the result was multiplied by f=3.6 to correct for signal compression. Charge uncertainties were estimated from the standard deviation of the voltage signal. Finally, object velocity was estimated using Eq. 3. Because all objects detected in the field settled slowly relative to the circuit response ($\tau _\text {RC} < \tau _p$), $\tau _p$ was measured from the time elapsed between the entrance and exit peaks. Velocity uncertainties were assessed by repeating the manual selection of entrance and exit times.

Following the approaches of Bonadonna et al. (2011); Bagheri et al. (2016), and Vecino et al. (2022), settling volcanic ash was simultaneously collected at the same locations on adhesive carbon tape and/or in dedicated trays for later characterisation at the University of Geneva (Fig. 3E). Carbon tape samples were fully scanned with a Keyence VHX7000 microscope at a resolution of 3.3 μm per pixel. Images were then binarised in Matlab using a threshold intensity selected for each sample. Grain-size distributions were obtained by measuring the circle-equivalent diameter of all individual particles identified in the binary images and converting particle counts to volume distributions following (Fries et al. 2023). Supplementary Table S2 reports sampling times and sites, grain-size measurements, and the classification of collected particles.

**Fig. 4:**
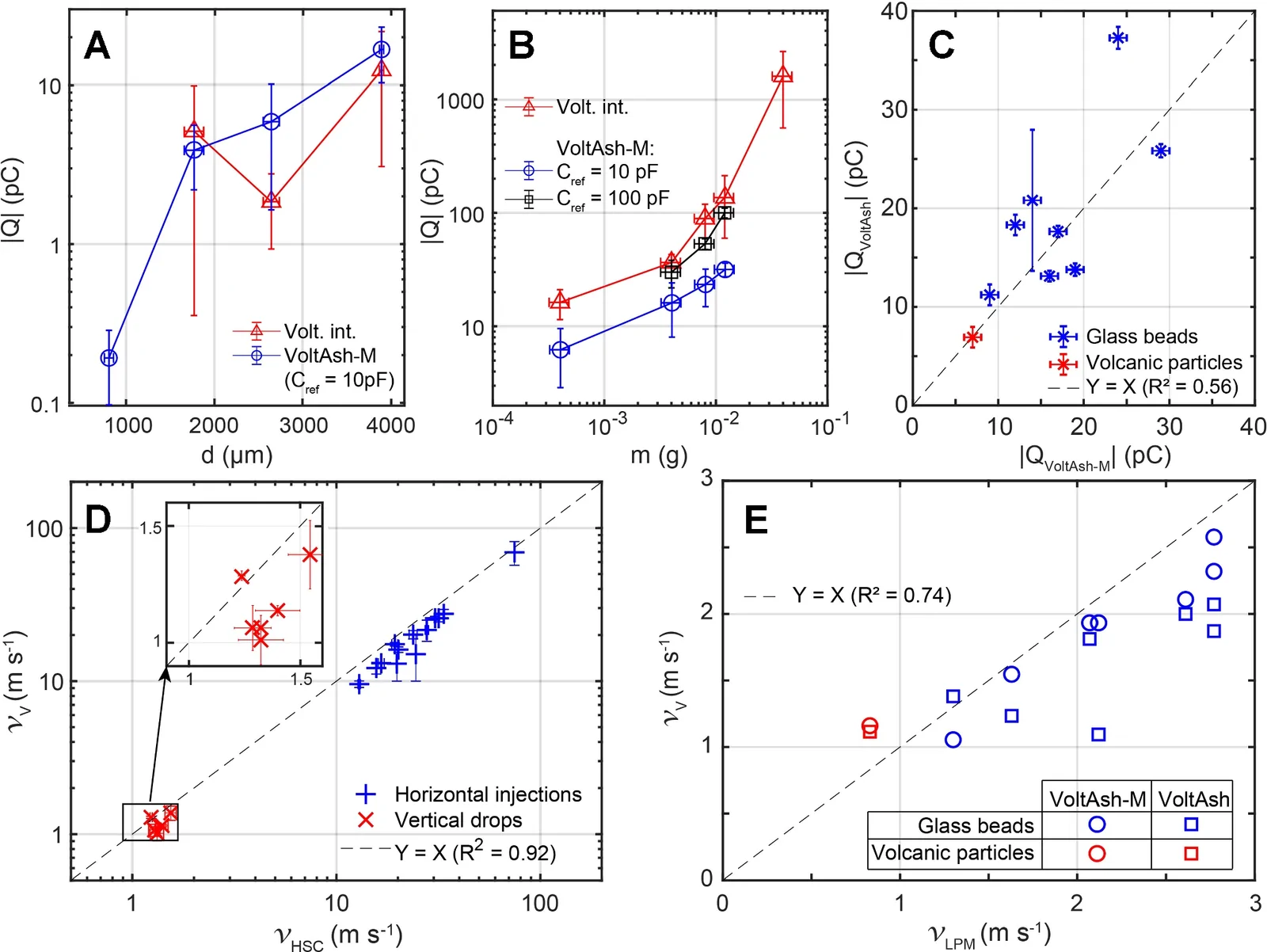
Laboratory validation of charge and velocity measurements. **A** Comparison of the electrical charges measured from voltage integration (labeled “Volt. int.”; Eq. 5) and with VoltAsh-M (the reference capacitance is indicated in the legend; Eq. 1) for individual particles (experiments Ex13–Ex19). **B** Electrical charges measured for given masses of fine particles using voltage integration and VoltAsh-M (Ex1–Ex12). **C** Particle charges estimated with VoltAsh-M and VoltAsh during paired measurements (Ex21; Fig. 2C). **D** Particle velocities within the TTFC obtained from the voltage signal ($v_\text {V}$) and high-speed videos ($v_\text {HSC}$). Blue markers correspond to particles injected horizontally with pressurised air, for which $\tau _\text {p} < \tau _\text {RC}$ (Ex13, Ex16, and Ex18; Fig. 2A). Red crosses correspond to particles dropped vertically without initial velocity, for which $\tau _\text {p} \ge \tau _\text {RC}$ (Ex20; Fig. 2B). The inset shows a close-up of the vertically falling particles. **E** Particle velocities estimated by VoltAsh-M and VoltAsh compared with LPM measurements (Ex21; Fig. 2C)

VoltAsh measurements were complemented by simultaneous LPM measurements to describe the temporal evolution of tephra fallout. LPM data were filtered following (Freret-Lorgeril et al. 2022) to discard false detections. Optical disdrometers are commonly used to characterise tephra fallout, notably at Sakurajima, where a permanent network monitors tephra sedimentation (Kozono et al. 2019; Takishita et al. 2024), and at Etna (Scollo et al. 2009; Marchetti et al. 2022). Here, the LPM provided the number of particles detected per minute, which was used as a proxy for temporal changes in sedimentation intensity. Although these measurements provide an independent constraint on fallout during the reported events, they are not directly comparable with VoltAsh data. The two instruments sampled different areas and differ in the parameters they are sensitive to: the LPM measures the size and settling velocity of particles larger than 0.15 mm, whereas VoltAsh detects charged objects crossing the TTFC. Information on LPM measurement times and locations, as well as the number, grain size, and velocity of detected particles, is provided in Supplementary Table S3.

## Results

### Laboratory tests

Electrical charge measurements obtained with VoltAsh-M and voltage integration were compared in dedicated laboratory experiments. All particles were negatively charged in this experimental setup, as expected for silica particles dispersed through an Al_2_O_3_ injector (Alois et al. 2018). Charges between 0.1 and 2000 pC were measured, covering values expected for volcanic ash particles, although volcanic ash aggregates may carry charges below the detection limit of the instruments (Pollastri et al. 2022). Figure 4A, B compares charges obtained by voltage integration (Eq. 5) and VoltAsh-M (Eq. 1). The measured charge increases with particle size and, for fine particles, with the mass released (Alois 2019). For individual particles larger than 1000 μm, both methods give similar results. VoltAsh-M operated with $C_\text {ref} = 10$ pF also detects lower charges carried by particles smaller than 1000 μm, which were not resolved by voltage integration. For fine particle masses, voltage integration gives values comparable to VoltAsh-M when $C_\text {ref} = 100$ pF, whereas measurements with $C_\text {ref} = 10$ pF are lower by up to a factor of five. The voltage peaks saturated for releases of 0.4 g, preventing charge estimation with VoltAsh-M for these tests.

Charge surface densities were calculated for individual particles from the measured charges. Both methods indicate charge surface densities between $10^{-8}$ and $10^{-6}$ C m$^{-2}$ for individual particles (Supplementary Fig. S6), in the range of values reported for volcanic ash (Pollastri et al. 2022).

VoltAsh and VoltAsh-M were also compared in paired charge measurements. Figure 4C compares dedicated experiments in which selected particles were measured consecutively with both versions of the instrument. Despite some variability, the charge estimates generally agree within a factor of two.

Particle velocities inferred from voltage signals were compared with independent high-speed camera measurements. Velocities obtained with VoltAsh-M, $v_\text {V}$, are generally consistent with those derived from high-speed videos, $v_\text {HSC}$, although they are slightly lower and associated with larger uncertainties (Fig. 4D). This agreement is observed for both $\tau _\text {p} < \tau _\text {RC}$, corresponding to high velocities, and $\tau _\text {p} \ge \tau _\text {RC}$, corresponding to lower velocities. Settling velocities measured with VoltAsh-M and VoltAsh also agree with LPM measurements, $v_\text {LPM}$, within a factor of two (Fig. 4E). During these experiments, particles were still accelerating between the successive measurements with VoltAsh, VoltAsh-M, and the LPM (Fig. 2C), which explains why $v_\text {V}$ is generally lower than $v_\text {LPM}$.

### Field measurements

VoltAsh detected multiple voltage peaks during volcanic ash fallout at both Sakurajima and Etna volcanoes. Most peaks correspond to slowly settling charged objects as exemplified in Fig. 3G.

#### Measurements at Sakurajima

**Fig. 5:**
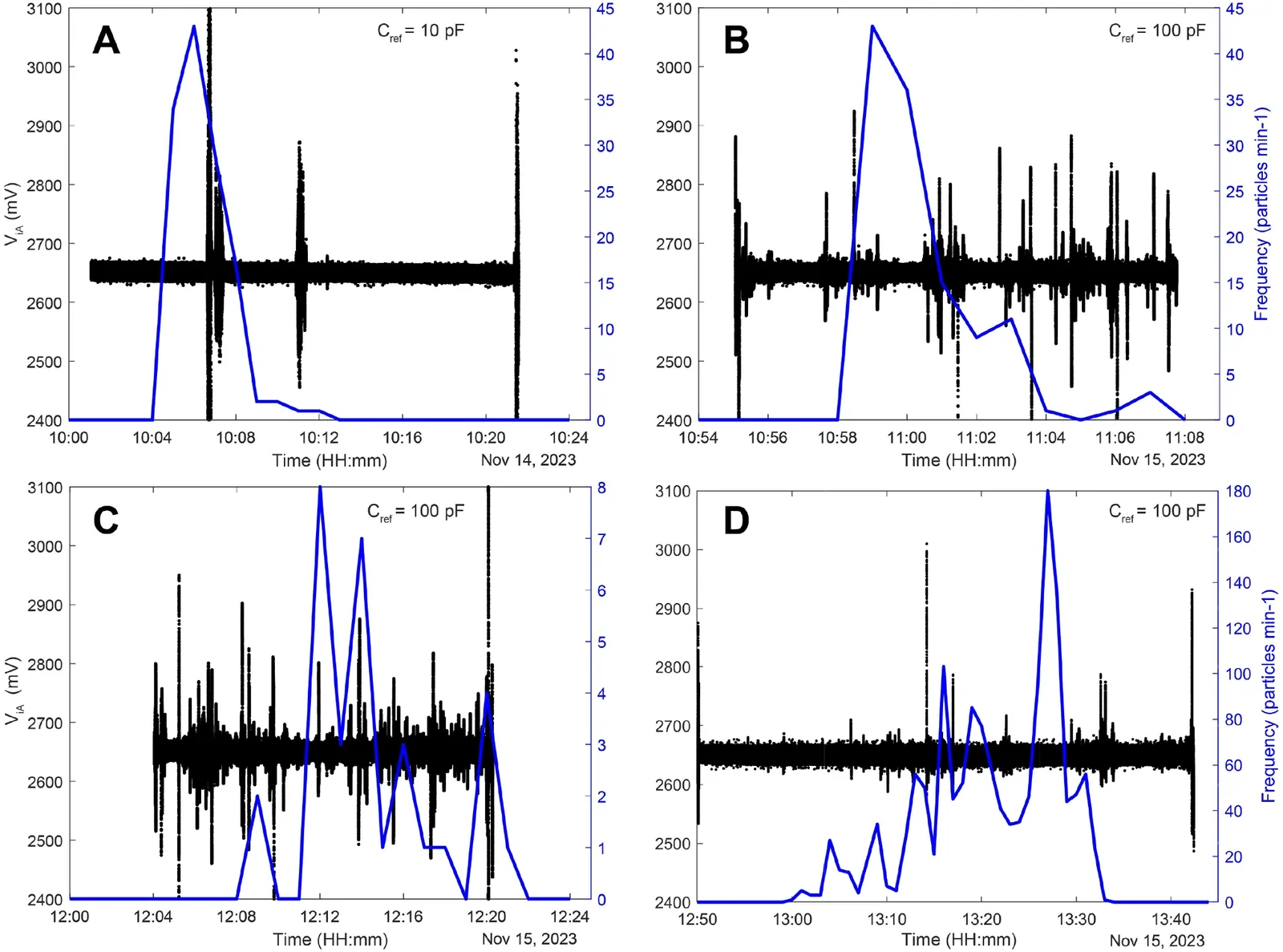
Voltage signals recorded by VoltAsh during field measurements **A** F6, **B** F8, **C** F10, and **D** F12 at Sakurajima volcano. VoltAsh measurements are compared with the frequency of detections by the LPM (blue solid lines, in number of objects detected per minute) at the same location

Numerous voltage peaks were recorded at Sakurajima, indicating that charged objects often settled through the TTFC (Fig. 5). Simultaneously, the LPM detected volcanic ash fallout at frequencies up to 180 particles min$^{-1}$. Because of the high detection frequency, successive voltage peaks commonly overlapped, inhibiting the accurate assessment of the electrical charge and velocity in this case, and resulting in underestimating the total number of charged particles. In total, 50 electrically charged objects could be analysed (Table 2).

**Fig. 6:**
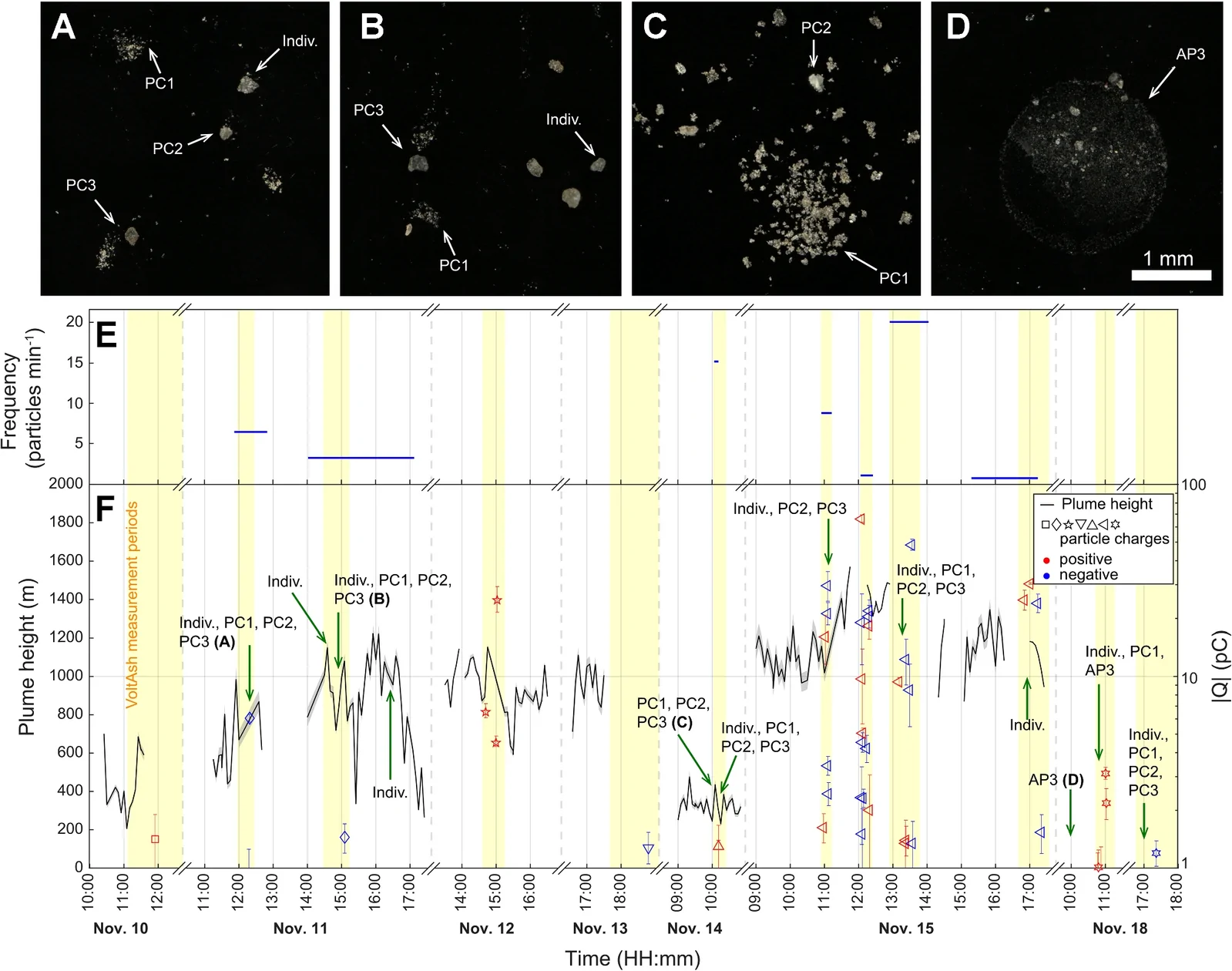
Temporal evolution of electrical charges detected at Sakurajima volcano. Objects sampled on adhesive carbon tape on **A** and **B** 11 November 2023, **C** 14 November 2023, and **D** 18 November 2023. Individual particles are indicated by “Indiv.”. Particle clusters are indicated by “PC1”, “PC2”, and “PC3” (Brown et al. 2012; Bagheri and Bonadonna 2016a). “AP3” indicates accretionary pellets, corresponding to liquid pellets (Brown et al. 2012). **E** Average LPM detection frequency. **F** Evolution of particle charge and plume height during the field campaign. Green arrows indicate sampling times and the type of particles observed. VoltAsh measurement periods are shown by yellow areas

The charged objects detected at Sakurajima carried both positive and negative bulk charges. The measured charges ranged from 0.7 to 200 pC. The voltage peaks are interpreted as resulting from individual volcanic ash particles or aggregates passing through the TTFC, as both types of objects were collected on adhesive tape during the field campaign (Fig. 6; Supplementary Table S2). The samples include individual particles and several types of aggregates, including ash clusters (PC1), coated particles (PC2), and cored clusters (PC3). Accretionary pellets, corresponding to liquid pellets (AP3), were only observed under rainy conditions on 16 and 18 November. Grain size analyses indicate median particle diameters below 500 μm, except for three samples collected on 16 and 18 November that contained AP3 particles (Supplementary Table S2). This is consistent with the LPM measurements, which indicate median diameters below 500 μm for all measurements at Sakurajima, with only sporadic detections of particles larger than 500 μm.

The largest number of analysed charges was obtained on 15 November, when plume height and fallout intensity were also highest. During the field campaign, plume heights measured from visible-wavelength cameras ranged from 200 to 1600 m above the vent, with the maximum reached on 15 November 2023 (Fig. 6). Short-term plume-height variations reflect intermittent ash emissions alternating with continuous ash venting and degassing. Adverse weather conditions prevented plume-height measurements on 18 November. Aggregates were sampled every day, despite generally weak ash emissions (Thivet et al. 2026). Electrical charges were also detected every day, but most were measured on 15 November, with 31 analysed objects. The most intense tephra fallout also occurred on that day, as shown by the LPM, which measured an average detection frequency of 20.3 particles min$^{-1}$ between 12:54 and 14:03 (Fig. 6E).

**Fig. 7:**
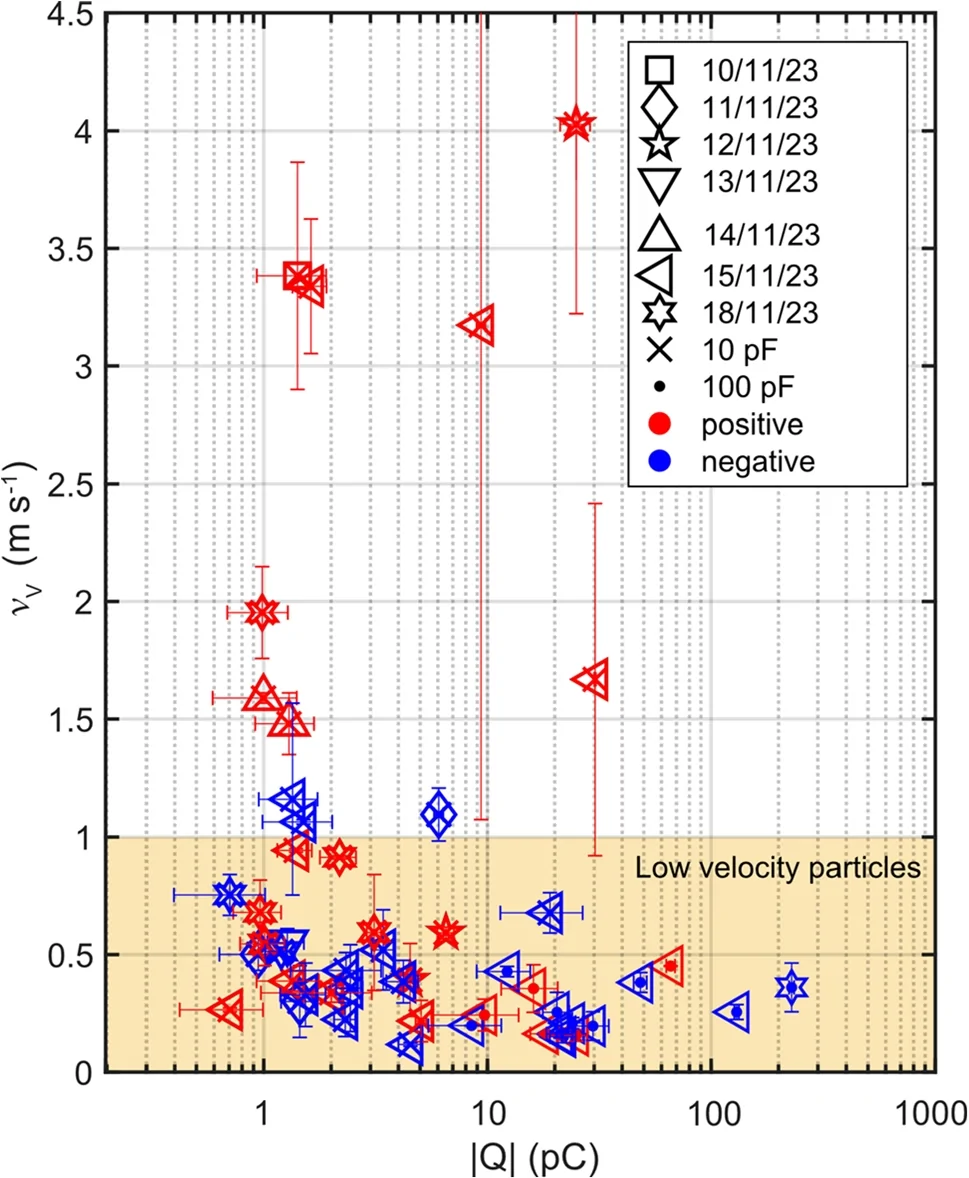
Fall velocity $v_\text {V}$ and bulk charge |*Q*| measured by VoltAsh for objects analysed during the field campaign at Sakurajima volcano. Marker shapes indicate the measurement day, and colours indicate charge polarity, with red for positively charged objects and blue for negatively charged objects. The reference capacitance is indicated by crosses and points for $C_\text {ref}=10$ and 100 pF, respectively. The full list of detected charges is provided in Supplementary Data S4

Settling velocities derived from analysing VoltAsh signals at Sakurajima range between 0.1 and 5 m s$^{-1}$ (Fig. 7). These values span the range previously measured at Sakurajima by Vecino et al. (2022); Freret-Lorgeril et al. (2022), and Pollastri et al. (2022), and are broadly consistent with simultaneous LPM measurements (Supplementary Table S3). Low settling velocities below 1 m s$^{-1}$ are inferred for 39 objects analysed here, slower than the velocities measured by Pollastri et al. (2022) for aggregates and their fragments.

**Fig. 8:**
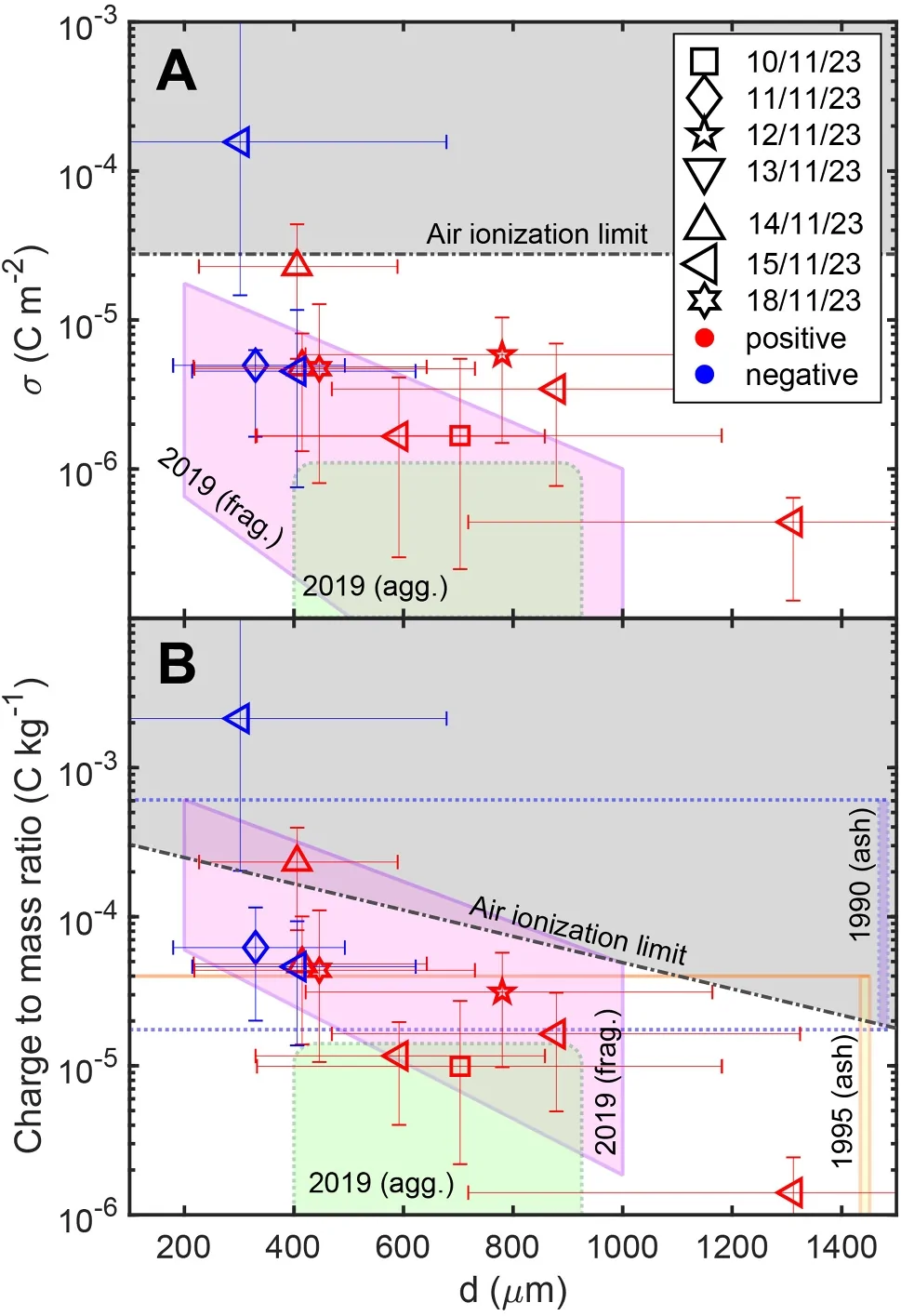
**A** Charge surface densities calculated for particles settling faster than 1 m s$^{-1}$ at Sakurajima volcano. **B** Charge-to-mass ratios calculated for the same particles. The air ionisation limit is shown by the dash-dotted line, and the region above this limit is shaded in grey. Results from Pollastri et al. (2022) are represented by the purple and green areas for individual fragments, “2019 (frag.)”, and aggregates, “2019 (agg.)”, respectively. Results from Gilbert et al. (1991) and Miura and Koyaguchi (2002) are indicated by the dotted blue line and orange line, respectively. Their charge-to-mass ratio estimates are represented by the blue vertical bar (“1990 (ash)”, Gilbert et al. 1991) and orange vertical bar (“1995 (ash)”, Miura and Koyaguchi 2002)

Charge surface density and charge-to-mass ratio were estimated for the objects detected at Sakurajima. Ranges of possible equivalent particle diameters were first inferred from the settling velocities detected with VoltAsh using the model of Bagheri and Bonadonna (2016b). This calculation was performed for densities of 750 and 2700 kg m$^{-3}$ and sphericities of 0.55 and 1, representing the range expected for aggregates and individual volcanic ash particles at Sakurajima (Freret-Lorgeril et al. 2022). The inferred diameters are consistent with particle sizes obtained from sample analyses and LPM measurements (Supplementary Tables S2 and S3). Particle masses were then calculated from these equivalent diameters by approximating the particles as spheres with densities of 750 and 2700 kg m$^{-3}$, allowing the charge-to-mass ratio to be estimated. Because the adopted density and sphericity ranges are broad, both the inferred diameters and derived charge surface density and charge-to-mass ratio have large uncertainties. Figure 8 only shows objects settling faster than 1 m s$^{-1}$, as slower objects yield charge surface densities above the maximum physically possible value for a charged sphere, corresponding to the air ionisation limit at 1 atm (Gilbert et al. 1991; Pollastri et al. 2022). Results including all particles are shown in Supplementary Fig. S7. For objects faster than 1 m s$^{-1}$, charge surface densities range from $10^{-7}$ to $10^{-3}$ C m$^{-2}$, and charge-to-mass ratios range from $10^{-6}$ to $4 \times 10^{-4}$ C kg$^{-1}$.

#### Measurements at Etna

**Fig. 9:**
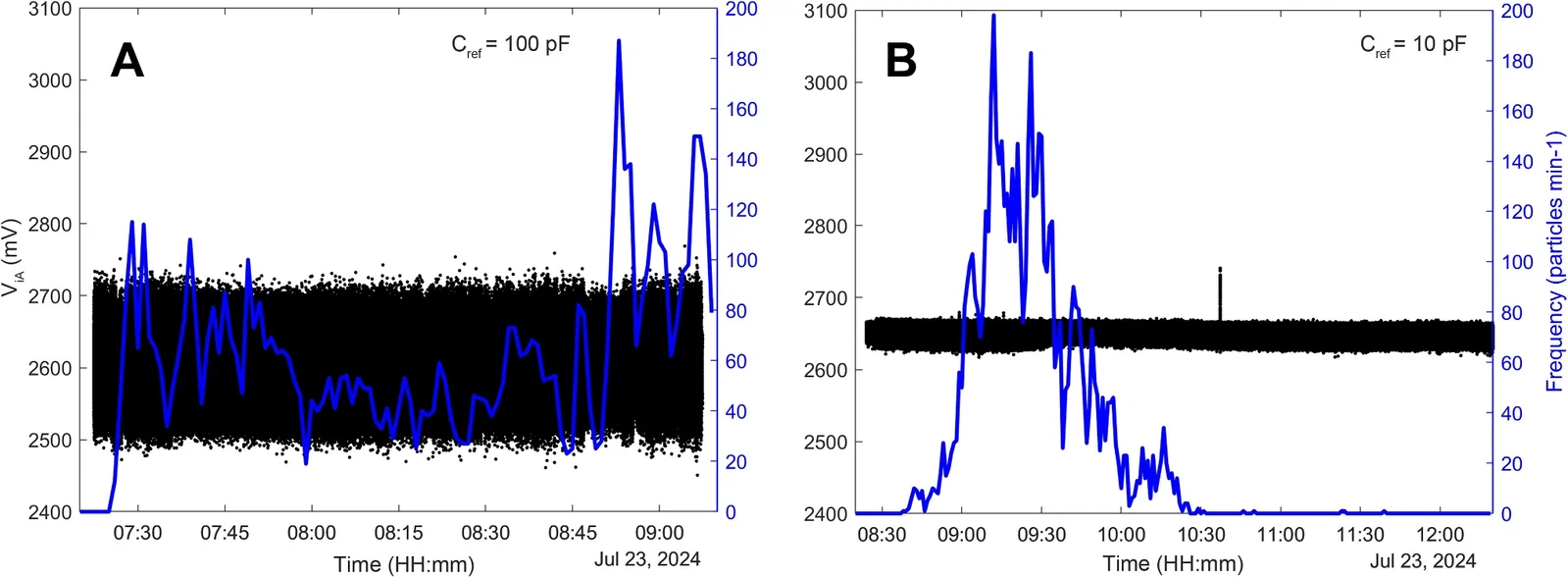
Voltage signals recorded by VoltAsh during field measurements **A** F19 and **B** F20 at Etna volcano. Unlike F20, F19 does not exhibit a discernible voltage peak attributable to the passage of a charged object. VoltAsh measurements are compared with the LPM detection frequency at the same location, expressed as the number of objects detected per minute blue solid lines

Voltage peaks were less frequently recorded at Etna than at Sakurajima, despite similar or higher LPM detection frequencies. The LPM detected up to 200 particles min$^{-1}$, with a temporal average of 61.2 particles min$^{-1}$ on 23 July 2024 (Fig. 9; Supplementary Table S3). Unlike at Sakurajima, no overlapping voltage peaks were detected. Five electrically charged objects could be analysed, with positive and negative charges ranging from 0.7 to 3.2 pC (Supplementary Data S5). Settling velocity could be estimated for two of these objects, with values of 0.25 and 4.83 m s$^{-1}$. These velocities fall within the broad range measured simultaneously by the LPM (Supplementary Table S3).

As no aggregates were sampled or observed during these measurements, the voltage peaks at Etna are interpreted as charged individual volcanic ash particles crossing the TTFC. Grain size analyses and LPM measurements indicate that the Etna particles were coarser than those sampled at Sakurajima, with diameters generally larger than 500 μm (Supplementary Table S2; Supplementary Fig. S8). Charge surface density for the two Etna particles are estimated at (8.5 ± 3.8) × $10^{-5}$ and (1.1 ± 0.5) × $10^{-6}$ C m$^{-2}$ assuming a density of 2800 kg m$^{-3}$, representative of dense basalt (Ferlito et al. 2017), and a sphericity of 1. The associated charge-to-mass ratios are (3 ± 1.1) × $10^{-3}$ and (4.5 ± 1.6) × $10^{-6}$ C kg$^{-1}$. The higher estimates of charge surface density and charge-to-mass ratio are obtained for the slow particle detected at 0.25 m s$^{-1}$ and all exceed the air ionisation limit at 1 atm (Supplementary Fig. S7).

## Discussion

Despite some instrumental limitations, our laboratory experiments and field measurements provide key insights into the electrical charges carried by volcanic ash.

### Advantages, disadvantages, and future developments of VoltAsh

Laboratory comparisons show that VoltAsh-M provides charge measurements broadly consistent with those derived from voltage integration. Measurements obtained with $C_\text {ref} = 10$ pF enable the detection of lower charges associated with smaller individual particles (Fig. 4A). For larger particle masses and higher charges, this configuration tends to yield lower estimates than the other methods, reflecting its higher sensitivity to small signals and earlier signal saturation. Using $C_\text {ref} = 100$ pF extends the measurable range towards higher charges, but reduces sensitivity to low-charge signals because of increased noise. In both configurations, signal saturation occurs for particle charges exceeding approximately 100 pC (Fig. 4B). Overall, VoltAsh-M measures charges between approximately 0.1 and 100 pC, with $C_\text {ref} = 10$ pF best suited to low charges, approximately 0.1–10 pC, and $C_\text {ref} = 100$ pF to higher charges, approximately 1–100 pC.

Laboratory measurements using VoltAsh-M and VoltAsh suggest that both versions provide comparable charge estimates for individually falling particles. Dedicated experiments, in which particles were measured consecutively with both versions, show agreement within a factor of two despite some variability (Fig. 4C). VoltAsh was not directly tested against voltage integration, nor with horizontally injected high-velocity particles. However, it uses the same charge-amplifier design as VoltAsh-M, so the main limitations identified for VoltAsh-M are expected to apply to VoltAsh.

Laboratory measurements also confirm that VoltAsh-M can estimate particle velocity from the voltage signal. The comparison with high-speed imagery shows good agreement for both fast ($\tau _\text {RC} \ge \tau _\text {p}$) and slowly settling ($\tau _\text {RC} \ll \tau _\text {p}$) particles (Fig. 4D). VoltAsh measurements were also compared with VoltAsh-M and LPM measurements obtained successively along the fall trajectory of particles (Fig. 2C). Because particles had not reached terminal velocity before these successive measurements, differences between instruments are expected, with the LPM generally giving higher velocities because it was positioned lower in the setup. Despite this, the three techniques provide comparable results (Fig. 4E).

Field measurements show that impacts with the inner wall of the TTFC are a major limitation for accurate estimates of the settling velocity. At Sakurajima, many objects had inferred velocities below 1 m s$^{-1}$, including 25 below 0.5 m s$^{-1}$ (Fig. 7; Supplementary Data S4). Such low velocities could correspond to highly porous volcanic ash aggregates, such as PC1 or PC3 types, as similar velocities were detected by the LPM and reported by Vecino et al. (2022) and Freret-Lorgeril et al. (2022). However, the calculated charge surface densities for these slow objects exceed the air ionisation limit, even when using broad ranges of density and sphericity to estimate particle size. This is unrealistic, as such high charge densities should be reduced by atmospheric discharges. These values therefore imply that the inferred settling velocities, and consequently the calculated equivalent diameters, are too low for the measured charges (Supplementary Fig. S7). The most likely explanation is that some particles decelerated after colliding with the inner wall of the TTFC. At Etna, where no aggregates were sampled, one object also had an inferred velocity below 0.5 m s$^{-1}$. Such a low velocity is unlikely for a dense spherical particle and may similarly reflect wall impact, although it may correspond to a highly porous or irregular particle. Adding a small camera operating at relatively high frame rate (such as the cost-effective Raspberry Pi Camera Module 3, similar to Thivet et al. 2025) would help identify particle trajectories, wall impacts, and differences between individual particles and aggregates.

VoltAsh-M and VoltAsh provide complementary configurations for laboratory and field measurements of particle charge. Their modular design allows the measurement range to be adapted by changing the reference capacitance: larger $C_\text {ref}$ values reduce saturation for highly charged particles, whereas smaller values improve sensitivity to weakly charged particles. The TTFC dimensions should also be adapted to particle concentration while maintaining an aspect ratio ≥1 (Weinheimer 1988). Under high particle concentrations, such as during intense tephra fallout, a smaller aperture reduces overlapping detections. Under low concentrations, larger apertures increase the probability of particle detection. VoltAsh-M is better suited to laboratory measurements because it provides short, triggered acquisitions that can be synchronised with complementary instruments such as high-speed cameras. However, field operation with VoltAsh-M was difficult, even when using a manually triggered 5 V step signal to synchronise measurements with high-speed videos. VoltAsh is more appropriate for field deployments because it operates automatically and semi-continuously, allowing longer measurements and simultaneous use of several instruments. In contrast, VoltAsh is less practical for targeted laboratory experiments because acquisition is interrupted by SD-card saving intervals.

VoltAsh-M and VoltAsh can also measure the velocity of charged particles over a wide range of conditions. Unlike optical disdrometers or high-speed cameras, their velocity measurements are not directly limited by particle size, but by particle charge. In principle, any particle can be detected if it carries a charge large enough to produce a measurable signal. This makes the method potentially useful for both slowly settling volcanic ash and faster particles, including particles moving within volcanic plumes. However, velocity estimates are affected if particles collide with the TTFC walls. For field measurements of settling volcanic ash, the method is therefore best suited to particles falling approximately vertically and weakly affected by winds. As mentioned above, the TTFC volume must be adapted to the sampling conditions, either to limit overlapping signals under high particle concentrations or to increase detections under low concentrations. Optimising this volume should increase the number of analysed particles, which is currently low compared with LPM detections. This could be further improved by developing automated signal-processing methods able to identify overlapping peaks produced by simultaneously detected particles.

Further technical developments could improve the efficiency and reliability of VoltAsh. First, using a different ADC, especially a bipolar ADC, could improve the signal-to-noise ratio and sampling frequency. Second, although the Arduino microcontroller is well suited to prototyping, a multi-threaded architecture would remove acquisition gaps caused by SD-card writing. Third, dedicated filtering and peak-detection algorithms could reduce manual processing for both charge and velocity measurements. A prototype based on a Raspberry Pi, an ADS1115 16-bit ADC, a simplified amplifier circuit, and a Raspberry Pi Camera Module 3 operating at 120 Hz is currently being developed to address these limitations (Supplementary Fig. S9).

Future versions of VoltAsh could be adapted for continuous long-term field measurements. A long-lasting installation would allow temporal variations in particle charge and settling velocity to be related to eruption dynamics and atmospheric conditions. Networks of low-cost VoltAsh sensors could also be deployed around volcanoes to investigate how the electrical charge evolves with distance from the source. Such developments are feasible because VoltAsh is inexpensive, simple to assemble, and easily reproducible. Beyond field monitoring, VoltAsh could also be used in laboratory experiments on particle electrification (cf. Watanabe et al. 2007; Méndez Harper and Dufek 2016) and aggregation mechanisms (e.g., 2017).

### The electrical charge of volcanic ash and aggregation processes

Field measurements at Sakurajima and Etna provide rare constraints on the electrical charge carried by volcanic ash particles and aggregates. At Sakurajima, charged objects were detected together with aggregates, allowing comparison with previous field measurements and discussion of possible links between electrification and aggregation. At Etna, fewer charged objects were detected, despite substantial ash fallout recorded by the LPM. Overall, only 55 charged objects were analysed, including five at Etna. This dataset provides rare *in-situ* observations of charged volcanic ash, but its limited size prevents a robust quantitative assessment of electrification and aggregation processes. The following discussion therefore focuses on qualitative interpretations supported by the available observations and previous studies.

#### Electrical charges and aggregation at Sakurajima

The charges measured at Sakurajima are generally comparable to previous measurements at the same volcano. In particular, VoltAsh detected objects carrying bulk charges from 0.5 to 100 pC, close to those previously measured for individual particles or fragments produced by aggregate breakup, which carried charges between 0.1 and 10 pC (Pollastri et al. 2022). However, Pollastri et al. (2022) reported lower charges for volcanic ash aggregates, typically between 0.01 and 1 pC. The charges measured here were therefore more likely carried by individual particles and fragments, rather than by aggregates. Complex signals potentially produced upon the fragmentation of aggregates did not allow to distinguish between individual fragments of different electrical polarities and to reveal the multipolar nature of aggregates.

The charge surface densities and charge-to-mass ratios estimated at Sakurajima are consistent with previous measurements for volcanic ash. For objects settling faster than 1 m s$^{-1}$, charge surface densities agree with values reported by Gilbert et al. (1991); Miura and Koyaguchi (2002), and Pollastri et al. (2022) (Fig. 8). The calculated charge-to-mass ratios, ranging from $10^{-6}$ to $4 \times 10^{-4}$ C kg$^{-1}$, also overlap with previous estimates at Sakurajima, although they are higher than the value of $4 \times 10^{-7}$ C kg$^{-1}$ measured at Mount Asama by Hatakeyama (1958). These values are particularly consistent with those measured for individual ash fragments by Pollastri et al. (2022), suggesting that many objects detected by VoltAsh were individual particles or aggregate fragments rather than intact aggregates.

The simultaneous detection of charged particles and aggregates supports a link between electrification and aggregation at Sakurajima. Electrostatic forces between oppositely charged particles can enhance collision efficiency and promote aggregation (Pollastri et al. 2021). In agreement with Pollastri et al. (2022), both positive and negative charges were measured, supporting the presence of oppositely charged particles during fallout. However, whilst (Pollastri et al. 2022) suggested that aggregation may neutralise the overall electric field of volcanic plumes through the binding of oppositely charged fragments, the present observations show that charges were frequently detected when aggregates were also sampled. Evidence suggests that both aggregation and electrification can be associated with the presence of fine ash and enhanced collisions in the plumes. This finding highlights the complex interdependence between charging and aggregation: strong charging promotes aggregation, while aggregation in turn gradually reduces the net charge of volcanic plumes. Testing this feedback would require further measurements tracking the temporal evolution of both particle charge and aggregation during eruptive events.

Furthermore, the measurements at Sakurajima demonstrate that volcanic ash can become substantially charged and aggregate even during weak eruptive activity. The November 2023 activity was dominated by low-altitude plumes generated by continuous ash venting and degassing, yet the measured charges are comparable to values reported after Vulcanian explosions. Most charges were nonetheless detected on 15 November 2023, when plume heights and fallout intensity were highest (Fig. 6). This suggests that stronger ash emission may enhance electrification, either through more efficient fragmentation and fracto-emission at the source or through higher particle concentrations promoting tribo-electrification (Vossen et al. 2024). This day also coincided with colder atmospheric temperature and volcanic plumes potentially overshooting the 0 °C isotherm (Supplementary Fig. S10), although the electrification of volcanic ash at Sakurajima is likely dominated by silicate particle charging, as the timescale of electrical activity at Sakurajima is short for ice nucleation to play a role as a major charging mechanism (Vossen et al. 2021). The frequent detection of charges during weak activity also indicates that intense explosions are not required to generate measurable ash electrification at Sakurajima.

The polarity of the detected objects may vary with size, although the limited dataset prevents robust quantification. Larger objects appear to carry predominantly positive charges (Fig. 8), consistent with size-dependent charging observed in natural tephra fallout and laboratory experiments (Hatakeyama 1958; Lacks and Levandovsky 2007; Cimarelli et al. 2014; Gaudin and Cimarelli 2019; Kikuchi and Endoh 1977). At the plume scale, size-dependent charging can promote charge and particle-size segregation (Miura and Koyaguchi 2002; Ichihara et al. 2023). At the particle scale, it can enhance aggregation by generating attractive forces between oppositely charged particles (James et al. 2002; Pollastri et al. 2021). However, the small number of measurements and the uncertainty on particle diameter prevent a detailed assessment of size-dependent charging in this study. Future versions of VoltAsh equipped with a synchronised camera, together with longer field deployments, would allow this process to be investigated more thoroughly. Drone-based deployments could also be used to examine charge segregation directly within volcanic plumes, as VoltAsh is sufficiently lightweight to be integrated into platforms such as AeroVolc (Thivet et al. 2026).

#### Differences in electrical charges observed at Sakurajima and Etna

Electrically charged objects were detected at both Sakurajima and Etna, supporting the widespread occurrence of ash electrification in volcanic plumes. However, only five charged objects were analysed at Etna, despite LPM detection frequencies similar to or higher than those measured at Sakurajima (Fig. 9). This suggests that ash particles were less frequently detected as charged objects at Etna. Several factors may explain this contrast, including differences in (1) magma composition, (2) eruptive style and fragmentation, (3) fine-ash abundance, (4) atmospheric conditions, and (5) distance from the source.

1. Magma composition may influence particle charging, although it is unlikely to explain the observed contrast alone. Recent Etna paroxysms produced mafic products with SiO_2_ contents of 46 to 50 wt% (Giuffrida et al. 2023), whereas Sakurajima ash is typically more silicic, with SiO_2_ contents of approximately 58 to 63 wt% (Yamanoi et al. 2008). These compositional differences may affect fragmentation behaviour and particle charging, but they are unlikely to be the dominant cause of the lower number of charged detections at Etna, where electrical activity has previously been observed during similar eruptions (Cimarelli et al. 2016, 2022).
2. Eruptive style and fragmentation mechanisms differed markedly between Etna and Sakurajima. Whilst Etna produced long-lasting lava fountains feeding sustained volcanic plumes, activity at Sakurajima was characterised by continuous ash venting interspersed with short-lived explosions. These differences may affect fracto-emission, which has been observed during the brittle fragmentation of pumice (James et al. 2000). Eruptive style and intensity also influence plume electrical activity, as shown during the 2021 Tajogaite eruption, where intense ash emissions associated with Strombolian activity and lava fountains generated stronger electrical discharges than mild ash emissions related to gas jetting (Vossen et al. 2024).
3. The abundance of fine ash likely played an important role in the observed difference between Sakurajima and Etna. Fine ash and polymodal grain-size distributions promote collisions through differential settling and favour the segregation of particles and charges according to aerodynamic properties (Cimarelli et al. 2014; Di Renzo and Urzay 2018). Variations in the amount of fine ash emitted may also influence the intensity of electrical discharges (Vossen et al. 2024). Because ash collected at Etna was substantially coarser than that collected at Sakurajima, the lower abundance of fine ash at Etna may have reduced both tribo-electrification and aggregation efficiency.
4. Atmospheric conditions may have modified ash electrification, but they do not fully explain the difference observed between the two volcanoes. High relative humidity can reduce the electrostatic charging of powders, including silica particles (Stern et al. 2019; Tkach et al. 2021), whereas ice-related processes can enhance volcanic plume electrification when plumes reach sufficiently cold atmospheric levels (Van Eaton et al. 2020; Prata et al. 2020; Van Eaton et al. 2022, 2023). At Etna, plumes reached the −10 $^{\circ }$C and −20 $^{\circ }$C isotherms, where ice-related charging processes may be favoured (Supplementary Fig. S10). In contrast, at Sakurajima, plumes generally remained between the 0 $^{\circ }$C and −10 °C isotherms when most charged objects were observed. Moreover, relative humidity was lower at Etna than at Sakurajima throughout the measurement period, which would not explain the lower number of charged detections at Etna if humidity primarily acted to dampen electrostatic charging. Hence, although atmospheric conditions may have contributed to modulating ash electrification at both volcanoes, their influence is equivocal. The combination of lower humidity and higher plume rise at Etna would be expected to promote, rather than inhibit, electrification, yet fewer charged objects were detected. This suggests that atmospheric conditions were probably secondary to eruptive style, fragmentation mechanisms, and fine-ash abundance in controlling the observed differences.
5. The distance from the source may influence charge detection by increasing the residence time of particles in the atmosphere. Particle charge can decay during transport through processes such as exposure to UV radiation (Ugolini et al. 2008) or the formation of conductive water layers on particle surfaces (de Lima Burgo et al. 2011). Measurements at Sakurajima were conducted within 5 km of the vent, whereas those at Etna were performed up to 15 km from the source. This longer transport distance may have favoured charge dissipation before measurement at Etna. However, the atmospheric residence time of particles was still on the order of tens of minutes at both volcanoes, whereas laboratory experiments show that airborne particles can retain charge for days to weeks (Méndez Harper et al. 2022). Distance from the source therefore cannot fully explain the lower number of charged detections at Etna.

Differences in eruptive style, fragmentation, and fine ash abundance provide the most likely explanation for the contrast between Sakurajima and Etna. Composition, transport distance, and atmospheric conditions may have contributed, but none of these factors alone explains the lower number of charged detections at Etna. The coarser grain size of volcanic ash produced at Etna suggests fewer fine particles available for collision-driven tribo-electrification and aggregation. In contrast, the finer ash fallout at Sakurajima likely favoured both particle charging and aggregate formation.

The observed differences in electrical charge may have influenced ash sedimentation at the two volcanoes. At Sakurajima, particle clusters were frequently observed, whereas only individual particles were detected at Etna (Supplementary Table S2). This contrast may result from both weaker particle charging and the lower abundance of fine ash at Etna. LPM measurements (Supplementary Table S3), tray samples (Supplementary Fig. S8), and adhesive-tape samples all confirm that Etna ash was consistently coarser than Sakurajima ash. These observations support the interpretation that stronger electrification and a higher abundance of fine ash at Sakurajima favoured aggregation, whereas coarser ash at Etna limited aggregate formation.

## Conclusion

The electrical charge and settling velocity of falling objects, including individual particles, aggregates and fragments, were measured simultaneously using VoltAsh, a cost-effective instrument combining a Through-Type Faraday Cage (TTFC) and a charge amplification circuit that enables automated measurements over extended periods. Laboratory experiments first validated the ability of VoltAsh to detect charged particles and estimate their settling velocities. These tests also highlighted the main limitations of the approach, including the need to select appropriate $C_\text {ref}$ values according to the expected charge range, uncertainties associated with particle collisions within the TTFC, and the absence of independent size measurements.

Field measurements were then conducted at Sakurajima and Etna volcanoes, leading to the following conclusions:

1. Simultaneous sampling revealed the presence of both aggregates and individual particles in the sedimentation from volcanic plumes at Sakurajima, whereas only individual particles were collected at Etna.
2. Charged objects were detected in low-intensity plumes at Sakurajima (<2000 m above vent; <3000 m a.s.l.), but most were recorded when the plume reached the maximum height observed during the field campaign. This suggests a link between eruption intensity, ash electrification and aggregation. The high charges and surface charge densities measured indicate that many detected objects were likely individual ash particles or aggregate fragments.
3. Most charged objects at Sakurajima were detected when plumes exceeded the 0 $^{\circ }$C isotherm. However, because Sakurajima plumes did not reach the −10 $^{\circ }$C isotherm, ice hydrometeors are unlikely to have been the dominant source of electrification. The measured charges are therefore interpreted as mainly resulting from silicate particle charging, including fracto-emission and tribo-electrification.
4. Fewer charged particles were detected at Etna than at Sakurajima, despite comparable particle detection rates during sustained, higher-intensity eruptions. This discrepancy likely arises from a combination of differences in eruptive style, fragmentation mechanisms, fine-ash abundance, particle size distribution, and the ability of particles to retain charge under the prevailing atmospheric conditions. Larger particles, as observed at Etna, are expected to have lower surface charge densities than smaller particles, which were more abundant at Sakurajima. Differences in humidity and freezing level may also have contributed, whereas differences in composition and in the distance between the source and measurement location are expected to have played a secondary role.
5. Aggregates were observed at Sakurajima but not at Etna. The greater abundance of fine ash at Sakurajima, together with the presence of charged particles, likely favoured aggregation. Our measurements suggest that electrification and aggregation are closely coupled processes, both controlled by the availability of fine particles, which in turn is controlled by eruptive style and intensity.

VoltAsh therefore provides a versatile and low-cost tool for investigating volcanic ash electrification and aggregation in both field and laboratory settings. It can be adapted for long-duration monitoring, distributed sensor networks, or low-cost measurements of ash settling velocity.

## Supplementary Information

Below is the link to the electronic supplementary material.

Supplementary file 1 (pdf 6295 KB)

## Data Availability

All data is available in supporting material and at: https://doi.org/10.5281/zenodo.17257359.

## Code availability

The code is available in supporting information.

## Materials availability

The material is made available on demand.
