# Supplementary material for: Measuring the electrical charge and settling velocity of volcanic ash with a new, cost-effective apparatus (VoltAsh) both in the laboratory and in the field
Source: Bull Volcanol. 2026 Aug 26;88(9):106. doi: 10.1007/s00445-026-02027-y (PMC13518462; doi:10.1007/s00445-026-02027-y)
Supplement: Supplementary file 1 [file 445_2026_2027_MOESM1_ESM.pdf]

Supplementary material for: Measuring the electrical charge and settling velocity of volcanic ash particles

Allan Fries*[1], Eduardo Rossi[1], Jonathan P. Merrison[2], Jens Jacob Iversen[2], Tatsushi Matsuyama[3], Frédéric Arlaud[1], Jonathan Lemus[1,4], Simon Thivet[1], Carolina Diaz-Vecino[1], Riccardo Simionato[1,4], Costanza Bonadonna[1]

[1] Département des Sciences de la Terre, University of Geneva

[2] Department of Physics and Astronomy, Aarhus University

[3] Department of Science and Engineering for Sustainable Innovation, Soka University

[4] Département d'informatique, University of Geneva

[*] allan.fries@unige.ch [corresponding author]

ORCiD (AF): 0000.0002.9060.1357
ORCiD (ER): 0000.0003.3749.5804
ORCiD (JPM): 0000.0003.4362.6356
ORCiD (JJI): 0000.0002.7879.6163
ORCiD (TM): 0000.000272719212
ORCiD (JL): 0009.0001.3644.0126
ORCiD (ST): 0000.0003.0836.6421
ORCiD (CDV): 0009.0002.4084.6484
ORCiD (RS): 0000.0002.1283.3136
ORCiD (CB): 0000.0002.2368.2193

TABLE OF CONTENT:

Supplementary Figures

- **Figure S1:** 3D view of the printed circuit board with the embedded charge amplifier.
- **Figure S2:** Schematic circuit diagram of the bipolar to unipolar converter.
- **Figure S3:** TTFCs used in field and laboratory experiments.
- **Figure S4:** Schematic view of the charge amplifier, bipolar to unipolar converter and connections with the Arduino board for the autonomous version.
- **Figure S5:** Celestron handheld digital microscope images of the particles used in laboratory experiments **A**. Ex1-Ex12, **B.** Ex14 and Ex18, **C.** Ex16**, D.** and Ex13 and Ex17.
- **Figure S6:** Surface charge densities and charge to mass ratios for particles in laboratory experiments.
- **Figure S7:** Surface charge densities and charge to mass ratios for all the particles detected during field experiments at Sakurajima and Etna volcanoes.
- **Figure S8:** Grainsize distribution of the sample collected in a tray at location E2.
- **Figure S9:** Raspberry Pi version of VoltAsh.
- **Figure S10:** Atmospheric parameters associated with analysed eruptions.

Supplementary Tables

- **Table S1:** GPS coordinates of the field locations
- **Table S2:** List of samples collected on adhesive tape.
- **Table S3:** Schematic view of the charge amplifier, bipolar to unipolar converter and connections with the Arduino board for the autonomous version.

Supplementary Data

- **Data S1:** Sketch for continuous voltage measurements and data save.
- **Data S2:** Laboratory experiments: description of the different tests, high speed videos; voltage signals associated with the experiments.
- **Data S3:** Field measurements: voltage signals recorded in the field.
- **DataS4:** Measurements of the particle charge and velocity at Sakurajima volcano.
- **DataS5:** Measurements of the particle charge and velocity at Etna volcano.

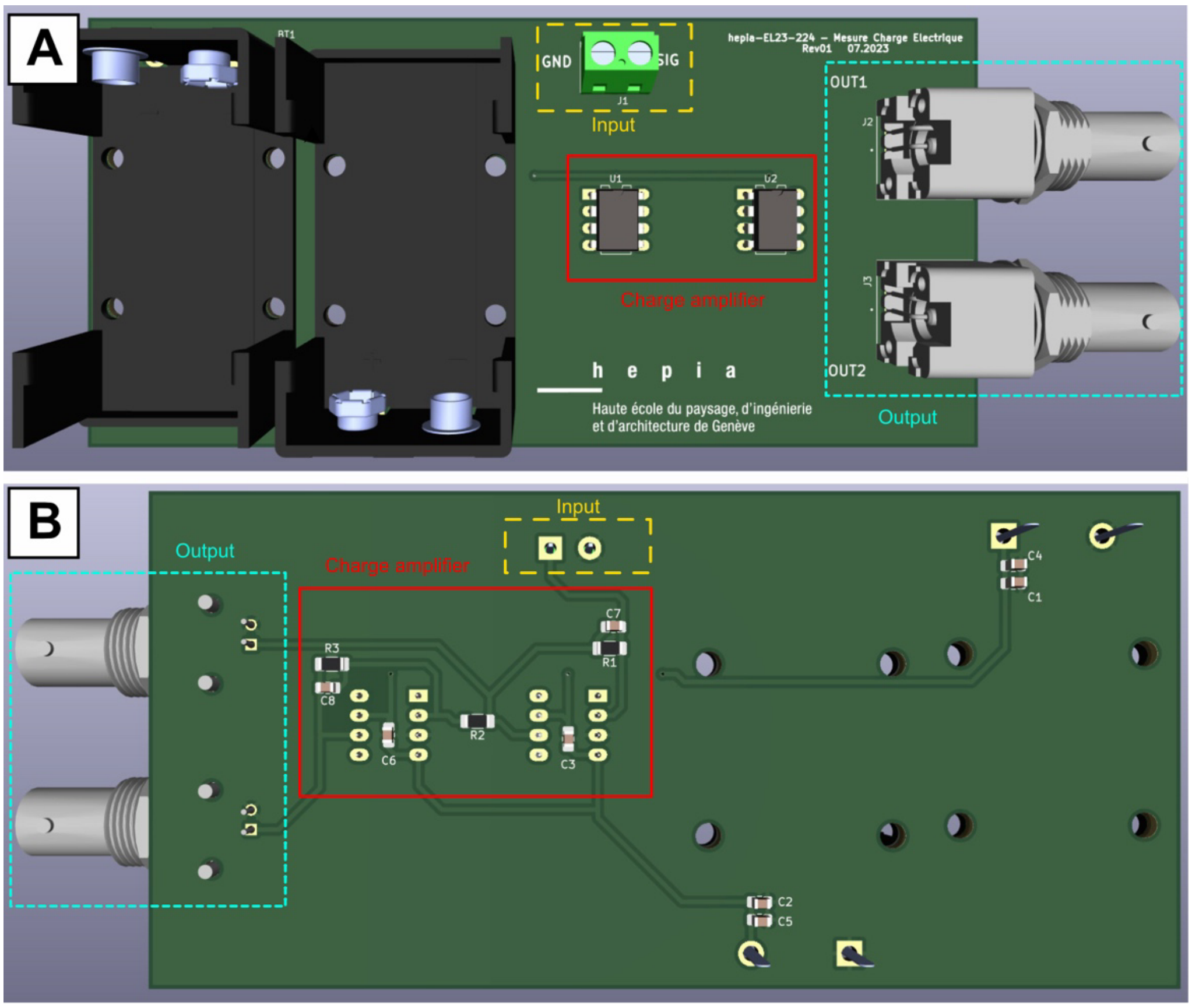

Figure S1 – 3D view of the printed circuit board with the embedded charge amplifier (VoltAsh-M). [A] Top view. [B] Bottom view. The input, charge amplifier and output are highlighted by the yellow dashed, red and blue dashed boxes. Condensers are identified by the letter "C" whilst "R" and "U" correspond to resistors and operational amplifiers, respectively.

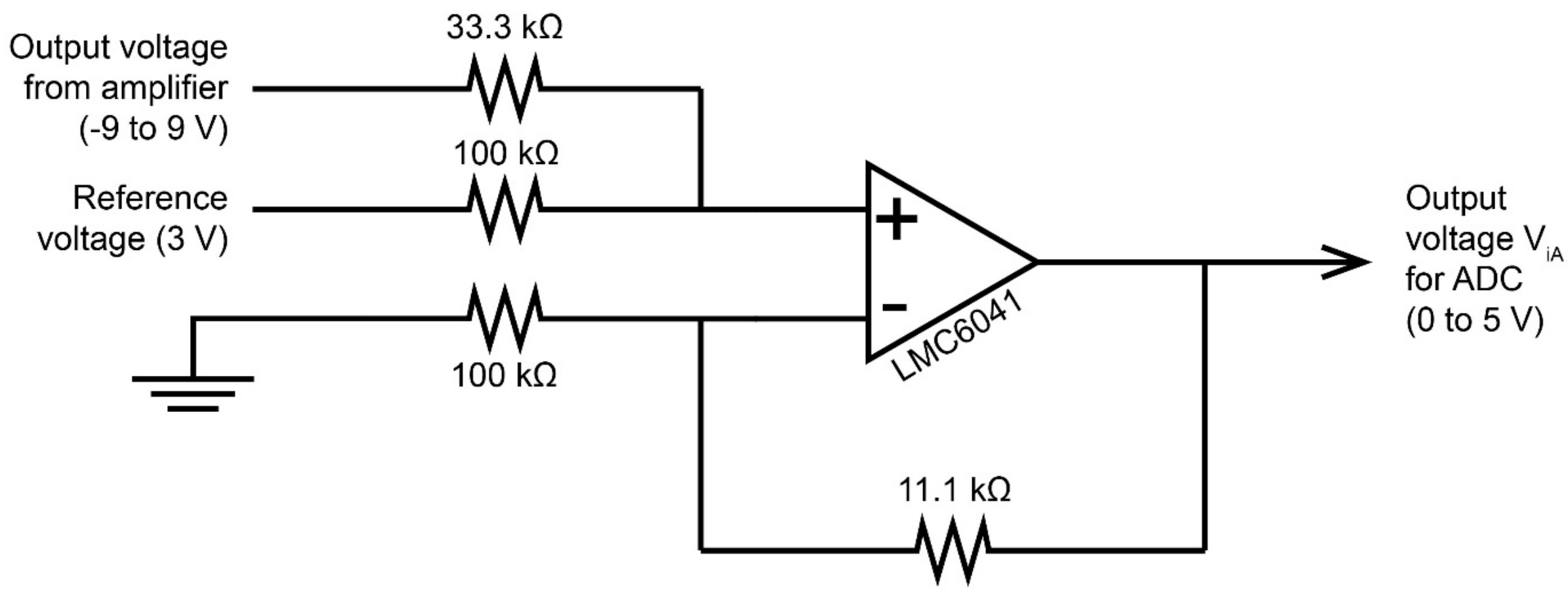

Figure S2 – Schematic circuit diagram of the bipolar to unipolar converter used in VoltAsh.

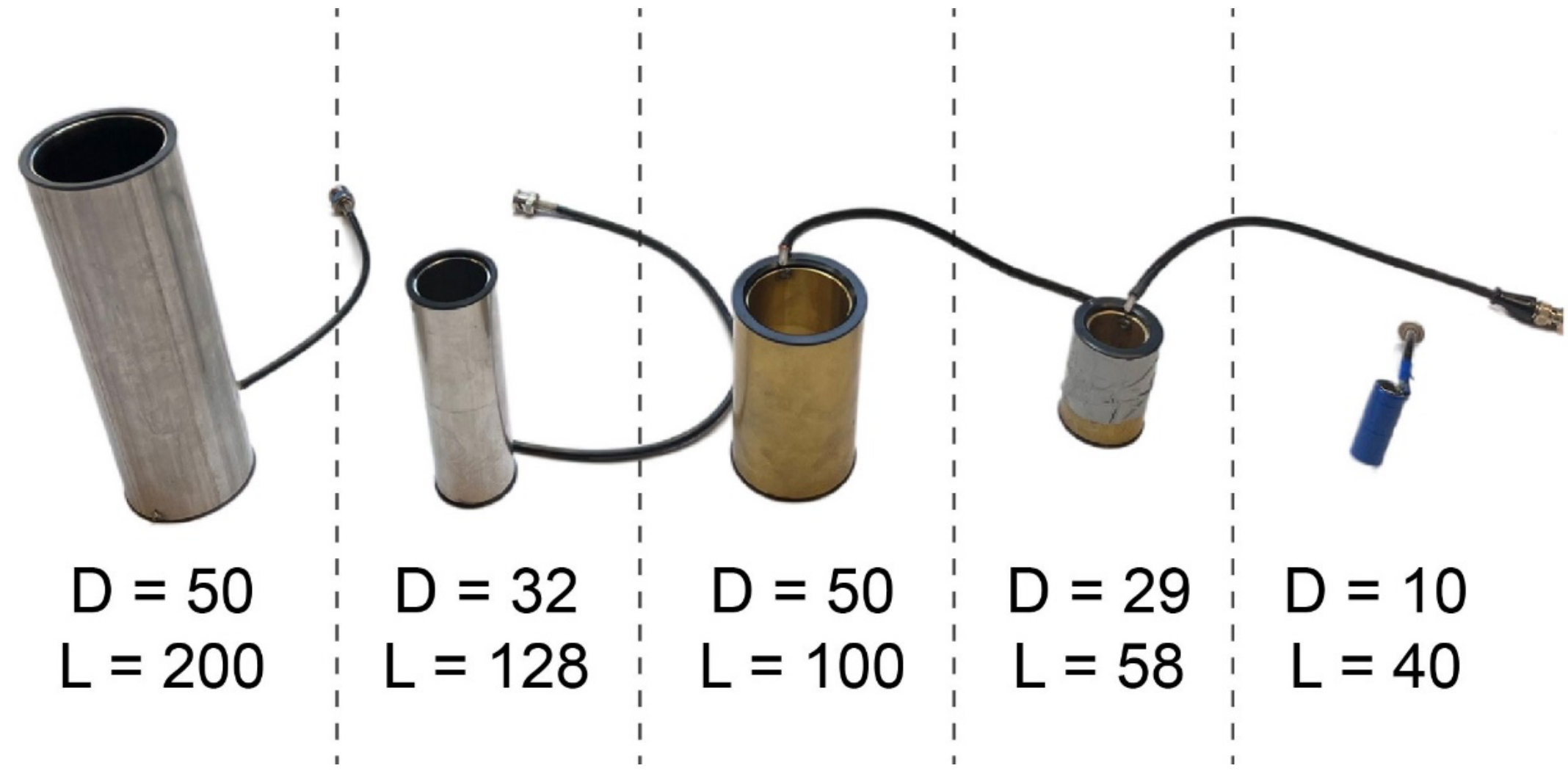

Figure S3 – TTFCs used in field and laboratory experiments, with corresponding aperture diameter D and lengths L given in mm.

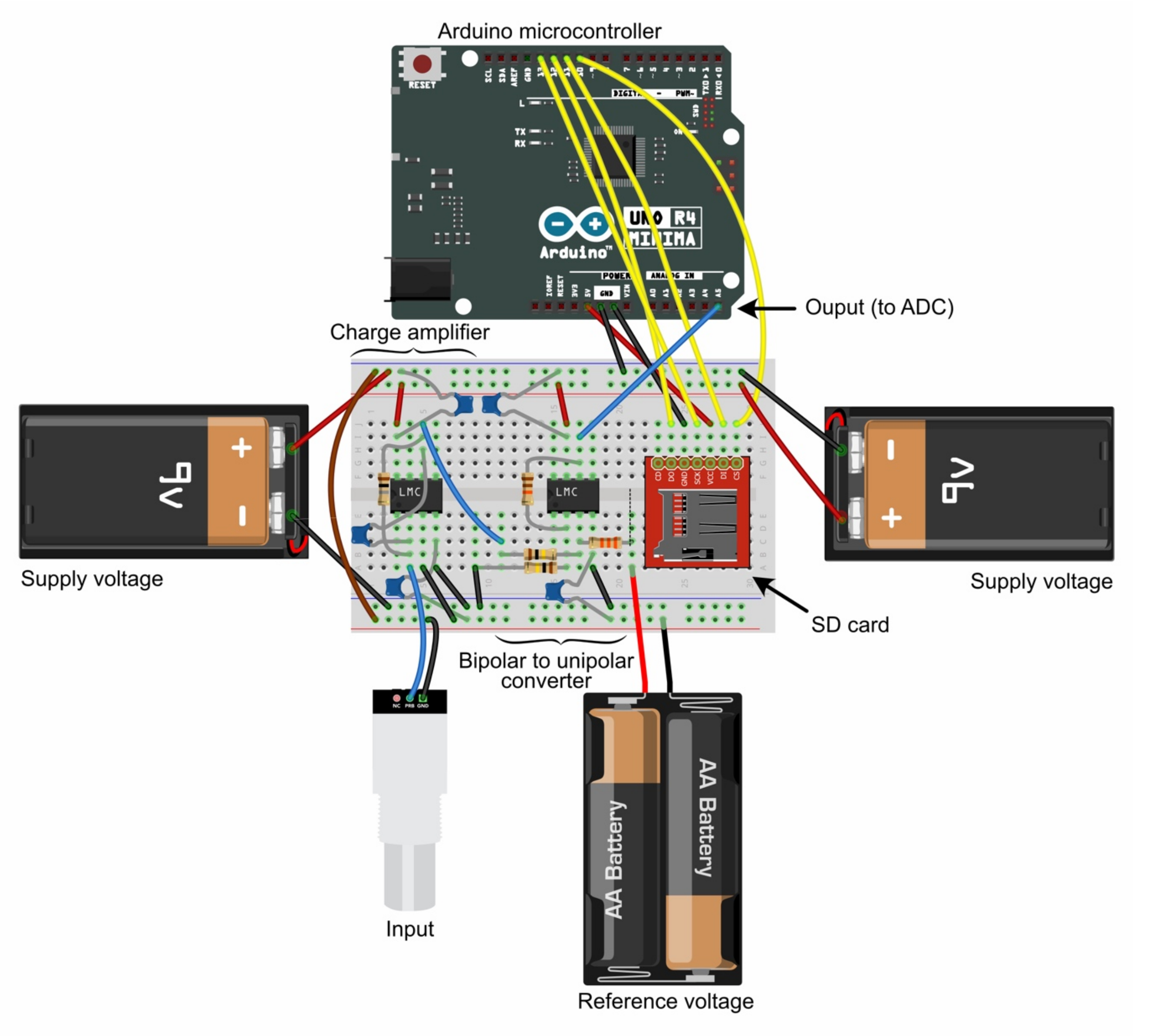

Figure S4 –Schematic view of the charge amplifier, bipolar to unipolar converter and connections with the Arduino board for VoltAsh.

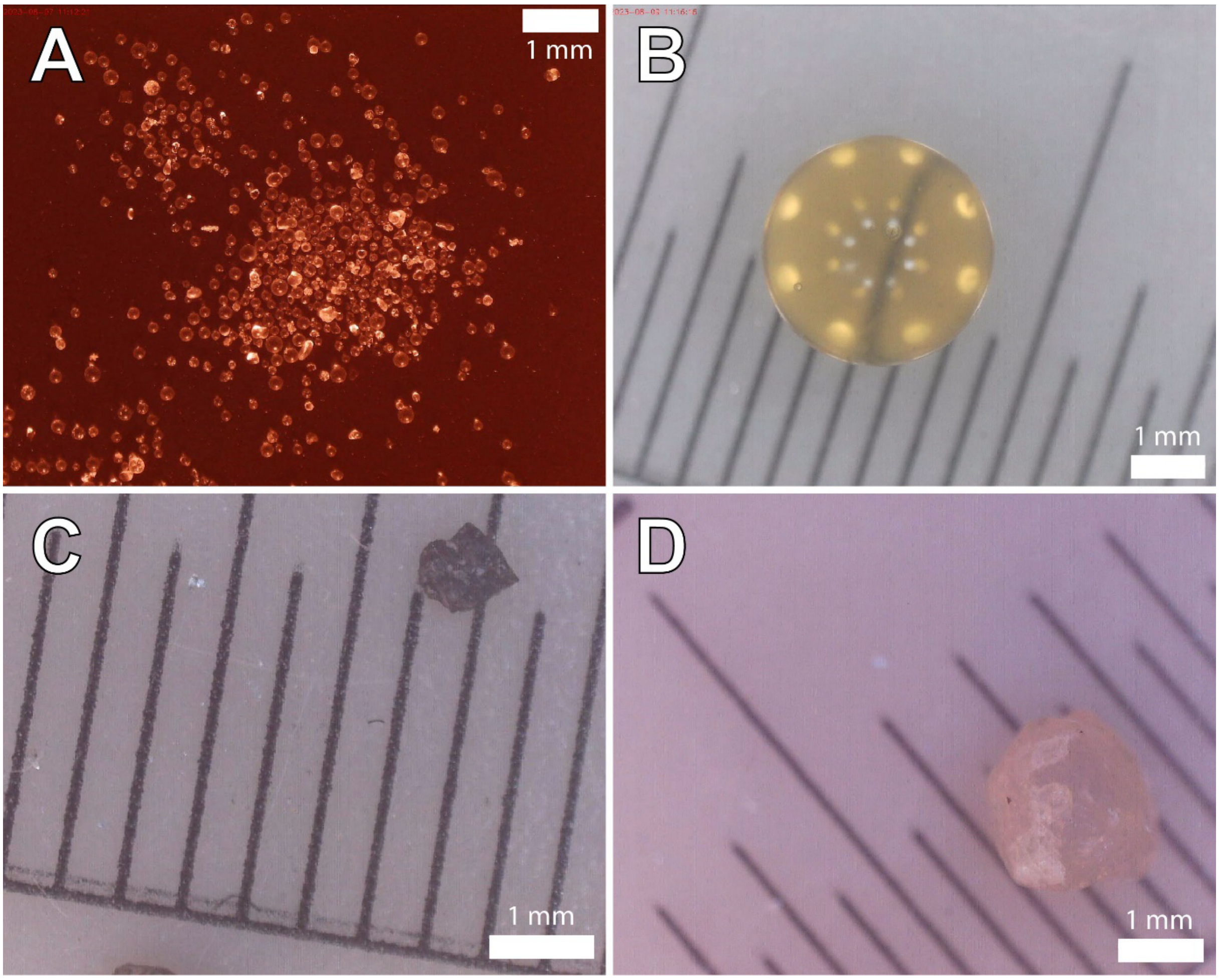

Figure S5 – Celestron handheld digital microscope images of the particles used in laboratory experiments A. Ex1-Ex12: fine glass beads, B. Ex14 and Ex18: large glass bead, C. Ex16: volcanic ash from Sakurajima volcano, D. and Ex13 and Ex17: quartz crystal.

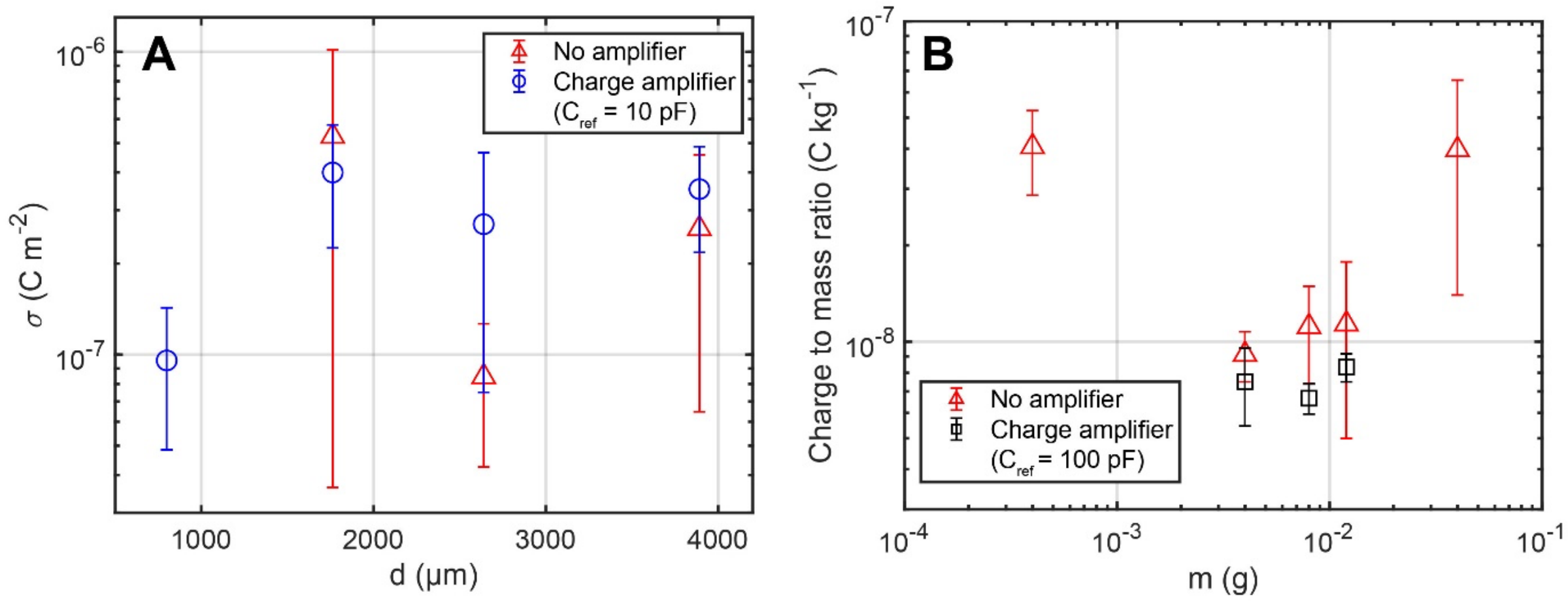

Figure S6 – A. Charge surface density σ calculated assuming that individual particles in laboratory experiments are spherical. B. Charge to mass ratios calculated for the release of particle masses in laboratory experiments.

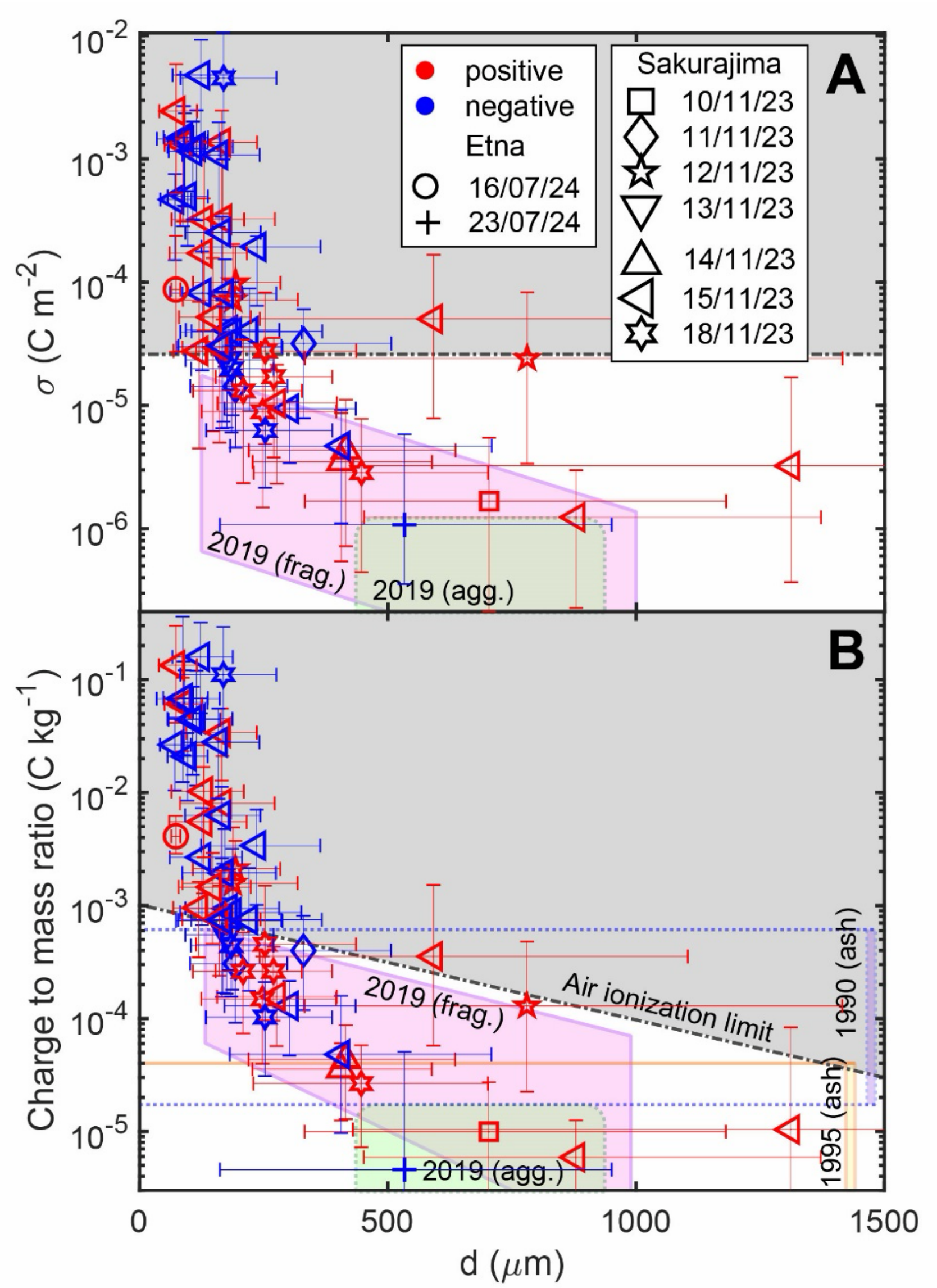

Figure S7 – A. Calculated surface charge density σ for particles from Sakurajima and Etna volcanoes assuming that the objects detected in the field are spherical. B. Charge to mass ratios calculated for the same objects assuming a density ranging from 750 kg m^{-3} to 2700 kg m^{-3} for Sakurajima, which covers the range from particle clusters to individual ash particles, and a density of 2800 kg m^{-3} for Etna. The range of possible particle diameters is calculated from the settling velocity using the equation of Bagheri and Bonadonna (2016) (see references in the main text), assuming a variation in sphericity of 0.55 to 1.

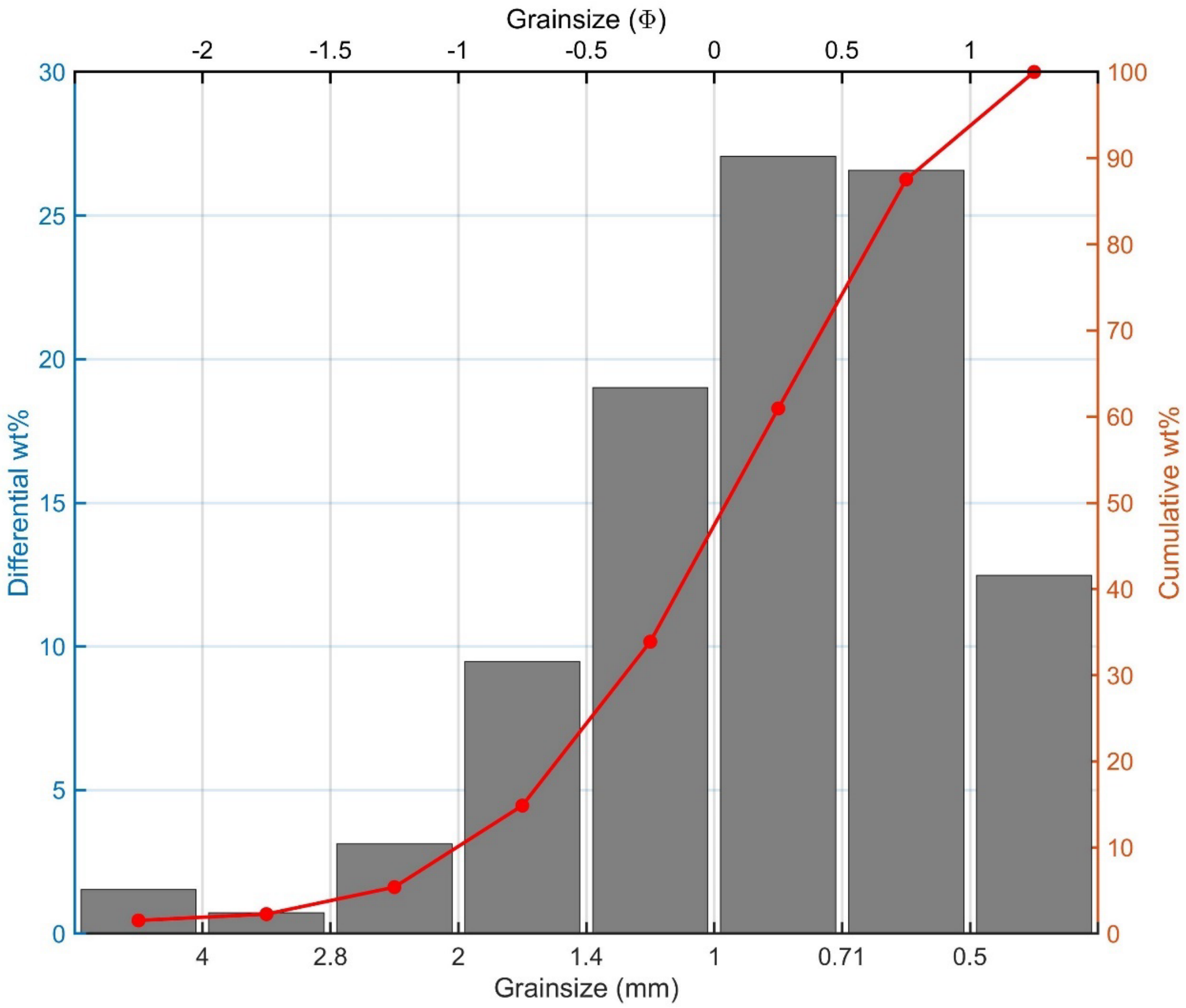

Figure S8 – Grainsize distribution of the sample collected in a tray at Etna volcano (location E2) from 07:25 to 09:07 (CET) on 23 July 2024. The distribution was obtained by sieving.

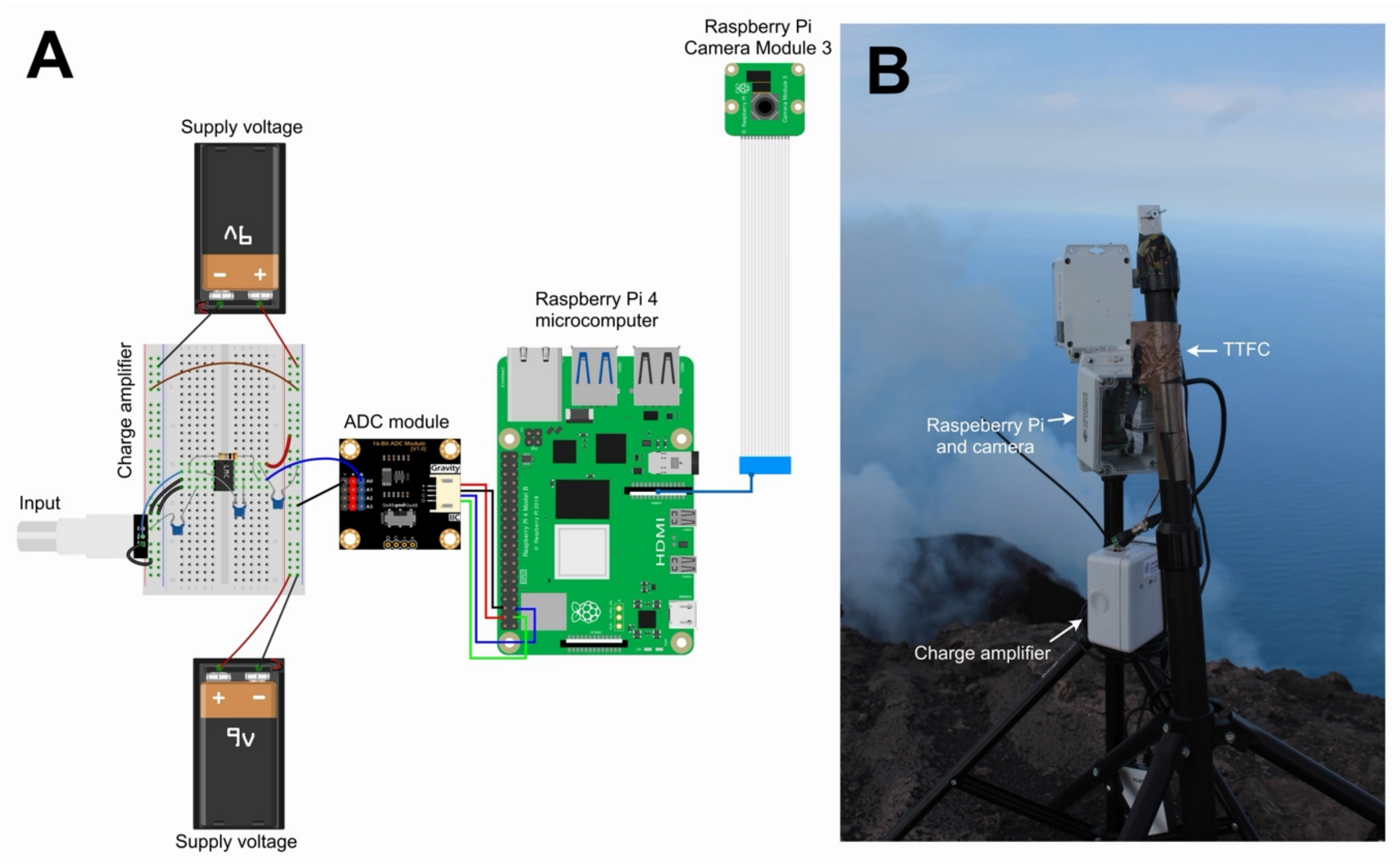

Figure S9 – Version of VoltAsh for Raspberry Pi microcomputer (VoltAsh-Pi). A. Sketch of the setup. B. VoltAsh-Pi in the field in September 2025 at Stromboli volcano, Italy.

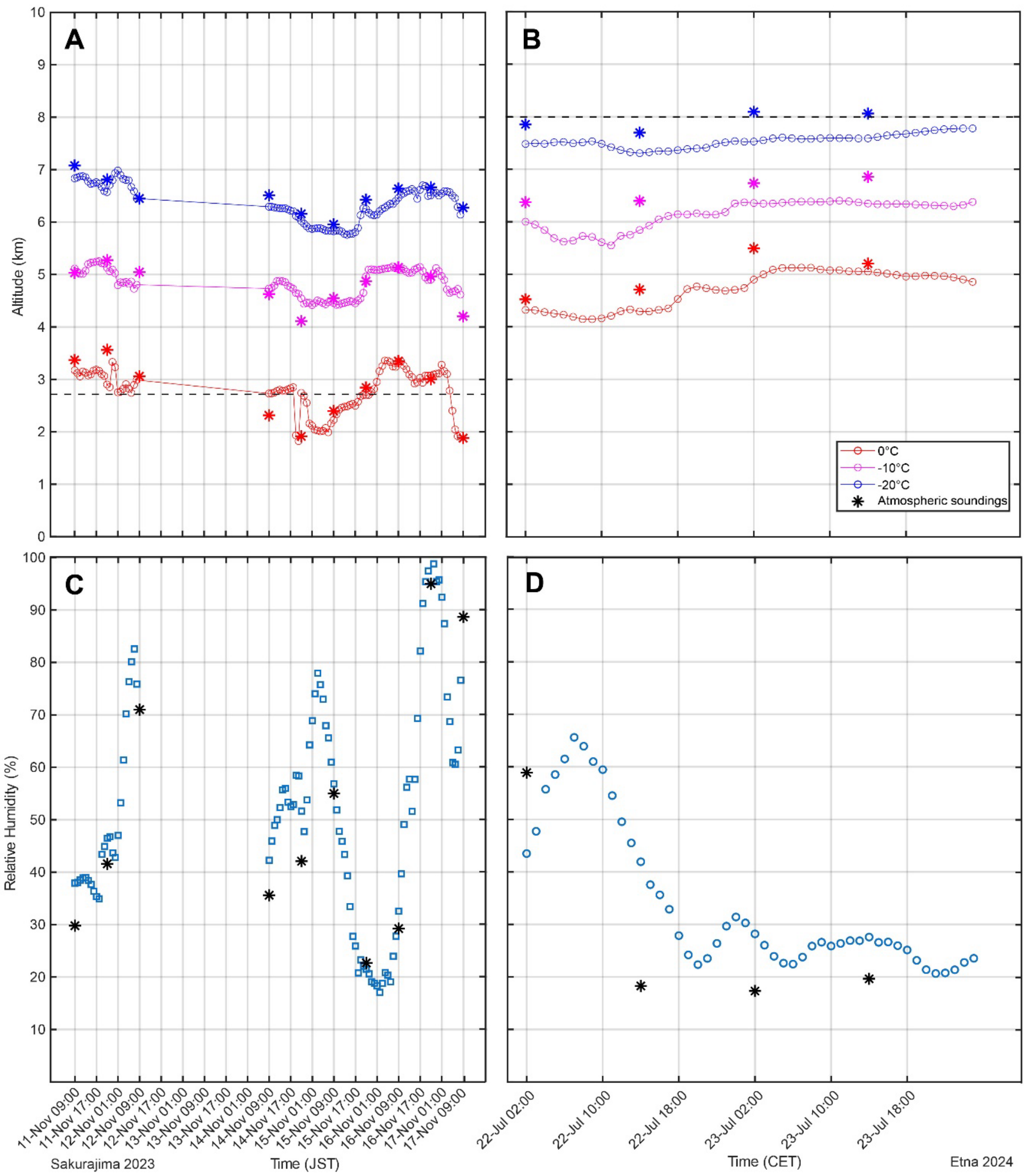

Figure S10 – Atmospheric parameters associated with analysed eruptions. Estimation of the altitude of the 0, -10 and -20°C isotherms at A. Sakurajima and B. Etna. The dashed lines indicate the maximum plume heights, as measured from calibrated video recordings (GVP 2024; Thivet et al. 2025). Evolution of the atmospheric relative humidity during the field campaigns at C. Sakurajima (average for altitudes characteristic of the plume height of 1 to 3 km above sea level) and D. Etna (average for altitudes of 3 to 8 km). Atmospheric data are sourced from ERA5 hourly reanalysis datasets (represented with squares and circles) and weather balloon soundings at 12 h intervals (represented with stars). The sounding data were obtained from the closest stations available of Kagoshima, approximately 10 km West of Sakurajima, and Trapani/Birgi, approximately 220 km West of Etna (University of Wyoming, Department of Atmospheric Science, Upper-air sounding data: http://weather.uwyo.edu/upperair/sounding.shtml).

Table S1 – Coordinates of the field sites as presented in Figure 3.

Location	Volcano	GPS coordinates
Sk1	Sakurajima	31°35'36"N 130°38'02"E
Sk2	Sakurajima	31°33'18"N 130°40'44"E
Sk3	Sakurajima	31°33'35"N 130°40'20"E
Sk4	Sakurajima	31°37'07"N 130°41'08"E
Et1	Etna	37°36'41"N 15°01'06"E
Et2	Etna	37°41'49"N 14°59'31"E

Table S2 – List of samples collected on adhesive paper. The location corresponds to the field locations indicated in Figure 3. Simultaneous field measurements of the electrical charge are indicated in the column "Field meas.". The date of sampling is given in DD/MM (2023; except for the sample at location E1 which was taken in 2024), and the start and end of sampling are in HH:mm (JST; except for the sample at location E1 which is in CET). The number of particles analysed on the adhesive tape is indicated in the column "Num part.". Main results are indicated in the following columns, including the median diameter "D_{50}", the sorting coefficient, and observations of the type of particles collected. "Indiv." indicates the presence of individual particles, PC1, PC2 and PC3 indicate the presence of particle clusters, and AP3 the presence of liquid accretionary pellets.

Location	Field meas.	Date	Start	End	Num. part.	D_{50} (µm)	Sorting (µm)	Observations
Sk2	F2	11/11	12:17	12:23	1598	181	42	Indiv., PC1, PC2, PC3
Sk2	F3	11/11	14:36	14:38	348	181	53	Indiv
Sk2	F3	11/11	14:55	15:00	4062	213	84	Indiv., PC1, PC2, PC3
Sk2	-	11/11	16:23	16:27	899	181	31	Indiv.
Sk4	F6	14/11	09:59	10:05	1729	452	243	PC1, PC2, PC3
Sk4	F6	14/11	10:05	10:20	1006	213	53	Indiv., PC1, PC2, PC3
Sk2	F11-12	15/11	13:00	13:05	1281	452	144	Indiv., PC1, PC2, PC3
Sk2	F11-12	15/11	13:11	13:15	2085	226	122	Indiv., PC1, PC2, PC3
Sk2	F11-12	15/11	13:17	13:22	2628	258	167	Indiv., PC1, PC2, PC3
Sk2	F11-12	15/11	13:25	13:30	3892	181	122	Indiv., PC1, PC2, PC3
Sk2	F13-14	15/11	16:51	17:00	765	320	235	Indiv.
Sk2	-	16/11	16:00	17:30	26997	515	362	AP3, PC1
Sk2	-	18/11	10:00	10:07	19052	1634	462	AP3
Sk2	F15-16	18/11	10:40	10:49	5352	710	352	Indiv., AP3, PC1,
Sk2	-	18/11	14:50	14:58	2307	213	137	Indiv., PC1, PC2, PC3
Sk2	F17	18/11	16:13	16:58	18208	151	96	Indiv., PC1, PC2, PC3
Et2	F19	23/07	08:10	08:18	409	536	197	Indiv

Table S3 – Disdrometer measurements, with the date indicated in DD/MM (2023; except for the sample at locations E1 and E2 which were taken in 2024) and the start and end times in HH:mm. Simultaneous field measurements of the electrical charge are indicated in the column "Field meas.". The number of particles detected is given in the column "Num. part.". The minimum, maximum and median diameters (i.e., D_{min}, D_{max} and D_{50}, respectively) are indicated in µm, as well as the sorting coefficient ("Sort."). The minimum and maximum velocities (i.e., v_{min} and v_{max}, respectively) are given in m s^{-1}.

Location	Field meas.	Date	Start	End	Num. part.	D_{min}	D_{max}	D_{50}	Sort.	v_{min}	v_{max}
Sk1	-	10/11	15:46	17:20	2060	150	1350	280	80	0.03	3.67
Sk2	F2	11/11	11:50	12:50	387	170	6800	270	45	0.06	2.69
Sk2	F3	11/11	14:00	17:08	671	150	8440	200	35	0.09	2.57
Sk4	F6	14/11	10:04	10:12	125	220	590	330	75	0.49	2.18
Sk2	F7-8	15/11	10:57	11:11	120	160	473	365	92	0.69	3.00
Sk2	F9-10	15/11	12:05	12:24	29	190	500	410	122	0.75	3.17
Sk2	F11-12	15/11	12:54	14:03	1400	150	420	320	100	0.11	4.13
Sk2	F13-14	15/11	15:16	17:13	162	160	320	230	70	0.17	3.20
Et1	F18	15/07	22:17	23:16	156	160	450	240	45	0.22	1.84
Et2	F19	23/07	07:25	09:09	6362	150	8390	610	210	0.07	6.69
Et1	F20	23/07	08:24	12:27	5927	150	4360	700	260	0.01	7.42

Data S1 – Sketch used to read voltage and save data to the SD card. Modified from the "Read Analog Voltage" example sketch in the Arduino Documentation (Last revision Nov. 20, 2022 by Killaship).

```
/*
  ReadAnalogVoltage: Reads an analog input on pin 0, converts it to voltage, and
prints the result to the Serial Monitor.Graphical representation is available using
Serial Plotter (Tools > Serial Plotter menu). Attach the center pin of a
potentiometer to pin A0, and the outside pins to +5V and ground.
  This example code is in the public domain.
  https://www.arduino.cc/en/Tutorial/BuiltInExamples/ReadAnalogVoltage
*/
// Library to communicate with the SD card
#include <SD.h>
#include <SPI.h>
// Count the files on the SD cards to add a new file with name dataXXXX with XXXX
the number of files
File root;
int fileCount = 0; // for counting files
int loopCounter = 0;
char filename[23]; // Declare filename in this scope
String folderPath = "/RESULTS/"; // Specify the folder path
String accumulatedData = ""; // Initialize a string to accumulate data
// Define the chip select pin for your SD card module
const int chipSelect = 10;
// the setup routine runs once when you press reset:
void setup() {
  // initialize serial communication at 115200 bits per second:
  Serial.begin(115200);
  delay(1000);
  // Initialize the SD card
  if (!SD.begin(chipSelect)) {
    Serial.println("SD card initialization failed!");
    return;
  }
 // List files in the root directory
  File root = SD.open(folderPath);
// Count the number of files at the root of the SD card:
  while (root.openNextFile()) {
    fileCount++;
  }
root.close();
  // Generate a unique filename the file for writing
  // Format the filename with the current counter value
  // Generate a unique filename with the folder path for writing
  snprintf(filename, sizeof(filename), "%sdata%04d.txt", folderPath.c_str(),
```

```
fileCount);
  // Open the file
  File dataFile = SD.open(filename, FILE_WRITE);
  if (dataFile) {
    Serial.println("File opened successfully");
    // Close the file
    dataFile.close();
  } else {
    Serial.println("Error opening the file");
  }
  // Change the number of bits for analog read
  analogReadResolution(14); //change to 14-bit resolution
    delay(2000);  //To avoid data lost, delay a period of time to wait for the
module to start
}
// the loop routine runs over and over again forever:
void loop() {
  loopCounter++; // Increment the loop counter
  // read the input on analog pin 0:
  int sensorValue = analogRead(A5);
  // Convert the analog reading (which goes from 0 - 16383) to a voltage expressed
in mV (0 - 5000mV):
  float voltage = sensorValue * (5.0 / 16383.0) * 1000;
  // Accumulate data in the string
  String data
= String(String(loopCounter) + ";" + String(millis()) + ";" + String(voltage) + "\n
");
  accumulatedData += data; // Add a newline between data points
  // Check if the loopCounter is a multiple of 750
  if (loopCounter % 750 == 0) {
    // Open the data file for writing
    File dataFile = SD.open(filename, FILE_WRITE);

    if (dataFile) {
      // Write the accumulated data to the file
      dataFile.println(accumulatedData);
      dataFile.close();
      accumulatedData = ""; // Clear the accumulated data
    } else {
      Serial.println("Error opening data file");
    }
  }
  delay(1);
}
```

Data S2 – Laboratory experiments: description of the different tests, high speed videos; voltage signals associated with the experiments.

List of contents:

- Laboratory_experiments.xlsx – Table containing the information on all laboratory experiments and repeat tests performed in this work.
- Voltage_signals
    - Data – Folder containing csv files for each repeat test, where the first column is the time (in s), the second column is the voltage reading for channel 1 (in V), and the third column is the voltage for channel 2 (in V; usually used for recording the timing at which particles are released). The txt files of Ex21 correspond to the signals recorded by VoltAsh (column 1 is the line number, column 2 is the time in ms, column 3 is the voltage in mV).
    - Figures – Folder containing oscilloscope screenshots of the voltage signals recorded during each repeat test.
- LPM_measurements – Folder containing LPM measurements for Ex21 repeat tests. Columns 3, 4 and 5 are respectively the time stamp, size and velocity of detected particles.
- High_Speed_Videos
    - Trajectories – Folder with the trajectories of particles emphasized in a single image.
    - Videos – High speed videos of the particles measured in laboratory experiments.

Data S3 – Field measurements: voltage signals recorded in the field.

Raw data measured in the field at Sakurajima and Etna. The txt files are composed of 3 columns, with column 1 the line number, column 2 the time of measurement in ms, and column 3 the voltage in mV.

DataS4 – Measurements of the particle charge and velocity at Sakurajima volcano.

List of contents:

- DataS4_Charges_Sakurajima.xlsx – Table containing the information on all charge and velocity measurements at Sakurajima.
- Individual_Charge_Peaks – Folder containing figures for each analysed voltage peak.

DataS5 – Measurements of the particle charge and velocity at Etna volcano.– Sketch

List of contents:

- DataS5_Charges_Etna.xlsx – Table containing the information on all charge and velocity measurements at Etna.
- Individual_Charge_Peaks – Folder containing figures for each analysed voltage peak.
